# Nuclear transport proteins: structure, function and disease relevance

**DOI:** 10.1038/s41392-023-01649-4

**Published:** 2023-11-10

**Authors:** Yang Yang, Lu Guo, Lin Chen, Bo Gong, Da Jia, Qingxiang Sun

**Affiliations:** 1 Department of Pulmonary and Critical Care Medicine, Sichuan Provincial People’s Hospital, School of Medicine, University of Electronic Science and Technology of China, Chengdu, China; 2 The Key Laboratory for Human Disease Gene Study of Sichuan Province and Department of Laboratory Medicine, Sichuan Academy of Medical Sciences & Sichuan Provincial People’s Hospital, University of Electronic Science and Technology of China, Chengdu, China; 3 Research Unit for Blindness Prevention of Chinese Academy of Medical Sciences (2019RU026), Sichuan Academy of Medical Sciences & Sichuan Provincial People’s Hospital, Chengdu, China; 4 Key Laboratory of Birth Defects and Related Diseases of Women and Children, Department of Pediatrics, West China Second University Hospital, State Key Laboratory of Biotherapy, Sichuan University, Chengdu, China; 5 Department of Pathology, State Key Laboratory of Biotherapy and Cancer Centre, West China Hospital, Sichuan University, and Collaborative Innovation Centre of Biotherapy, Chengdu, China

**Keywords:** Cell biology, Oncogenesis, Neurological disorders, Molecular biology, Structural biology

## Abstract

Proper subcellular localization is crucial for the functioning of biomacromolecules, including proteins and RNAs. Nuclear transport is a fundamental cellular process that regulates the localization of many macromolecules within the nuclear or cytoplasmic compartments. In humans, approximately 60 proteins are involved in nuclear transport, including nucleoporins that form membrane-embedded nuclear pore complexes, karyopherins that transport cargoes through these complexes, and Ran system proteins that ensure directed and rapid transport. Many of these nuclear transport proteins play additional and essential roles in mitosis, biomolecular condensation, and gene transcription. Dysregulation of nuclear transport is linked to major human diseases such as cancer, neurodegenerative diseases, and viral infections. Selinexor (KPT-330), an inhibitor targeting the nuclear export factor XPO1 (also known as CRM1), was approved in 2019 to treat two types of blood cancers, and dozens of clinical trials of are ongoing. This review summarizes approximately three decades of research data in this field but focuses on the structure and function of individual nuclear transport proteins from recent studies, providing a cutting-edge and holistic view on the role of nuclear transport proteins in health and disease. In-depth knowledge of this rapidly evolving field has the potential to bring new insights into fundamental biology, pathogenic mechanisms, and therapeutic approaches.

## Introduction

Eukaryotic cells store genetic material in the nucleus and separate it from other cellular components using a double-layered membrane called the nuclear envelope (NE). This compartmentalization allows for complex and specialized cellular activities while simultaneously posing challenges for the exchange of materials between the nucleus and the cytoplasm. The vast majority of material exchange occurs through nuclear pore complexes (NPCs), which form channels in the NE. The transport of molecules into and out of the nucleus determines the subcellular localization of many macromolecules, e.g., transcription factor, and is fundamental in the regulation of gene expression, cell division, and other critical cellular functions.^1^

To facilitate nuclear transport (or nucleocytoplasmic transport), human cells dedicated approximately 60 different proteins to constitute the nuclear transport system (NTS).^2^ Each of these nuclear transport proteins (NTPs) has a defined function. One component of the NTS is the NPC, which is formed by nucleoporin proteins and presents a selective barrier to free diffusion of macromolecules into and out of the nucleus.^3^ The karyopherin family proteins, such as importins, exportins, and bidirectional transporters (biportins), act as molecular shuttles to transport macromolecules through NPCs.^4^ The small GTPase protein Ran and accessory factors regulate the transport direction and accelerate transport speed.^5^ In addition, proteins involved in nuclear transport have been demonstrated to have nontransport functions, including roles in mitosis, regulation of transcription, and regulation of biomolecular condensates.^6,7^ It is often possible to distinguish the contribution of canonical and noncanonical functions of NTPs in a certain cellular process. Due to these important cellular functions, dysregulation of the NTS is implicated in a range of human diseases, including cancer, neurodegenerative disorders, and viral infections.^8,9^

While different NTPs are often tightly linked in many cellular processes, most previous reviews have not included all three NTP classes: karyopherins, nucleoporins, and Ran system proteins. A holistic view of the NTS could facilitate the understanding of relevant phenomena and guide the development of therapies for diseases. In this review, we will explore the structure, function, and disease relevance of individual NTPs, with a focus on their interaction mechanism and networks, underlying principles, and potential therapeutic targets. We will draw on key foundations dating back decades as well as recent literature to summarize and discuss the vast body of knowledge acquired, and hopefully bring new perspectives to future research.

## Retrospective summary of research milestones

**Fig. 1:**
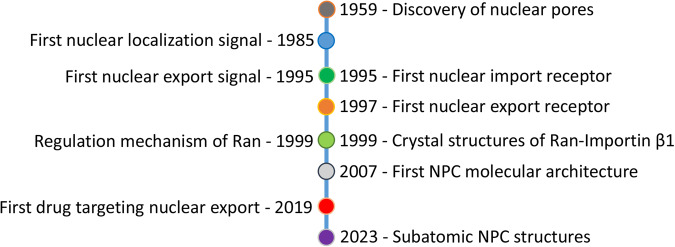
Research milestones in the field of nuclear transport

Due to their large size, cylindrical nuclear pore complex penetrating the nuclear envelope were discovered under electron microscopy as early as 1959 (Fig. 1).^10,11^. The first nuclear localization signal, which localizes yeast ribosomal protein L3 to the nucleus, was reported in 1985.^12^ Although the existence of nuclear import receptors was suspected at the time, first import receptor, p97 (now known as importin β1), was identified about a decade later.^13,14^ Shortly after, the first nuclear export signal and the first nuclear export receptor CRM1 were also identified.^15–17^ Ran-mediated regulation of nuclear cytoplasmic transport was discovered slightly earlier, but its role in nuclear transport was not well-understood until 1999.^18^ In the same year, the report of Ran-Importin β1 crystal structures marked that the field of nuclear transport entered the structural era.^19,20^ Using a collection of biophysical and proteomic techniques, the first molecular architecture of yeast NPC was built in 2007.^21^ Thereafter, with the development of cryo-EM and cryo-ET (electron tomography), the resolution of NPC structures has gradually increased to the current subatomic level.^22,23^ In the 1990s and 2000s, it was demonstrated that these NTS proteins also regulate mitosis, biomolecular condensates, and gene transcription, and are therefore implicated in various human diseases, including cancers, although many of the underlying mechanisms have not been revealed until recently.^24–27^ A drug targeting CRM1 was approved to treat two types of relapsed or refractory hematological cancers in 2019.^28^

## Components of the nuclear transport system

The 60 NTPs can be classified into three groups: the nucleoporins that form the nuclear membrane-embedded NPC, the karyopherins that bind and ferry cargoes through NPCs, and the Ran system proteins that determine transport directionality and efficiency.^2^ In this section, we will discuss the structure and function of each NTP class.

### Nucleoporins that form the nuclear pore complex

**Fig. 2:**
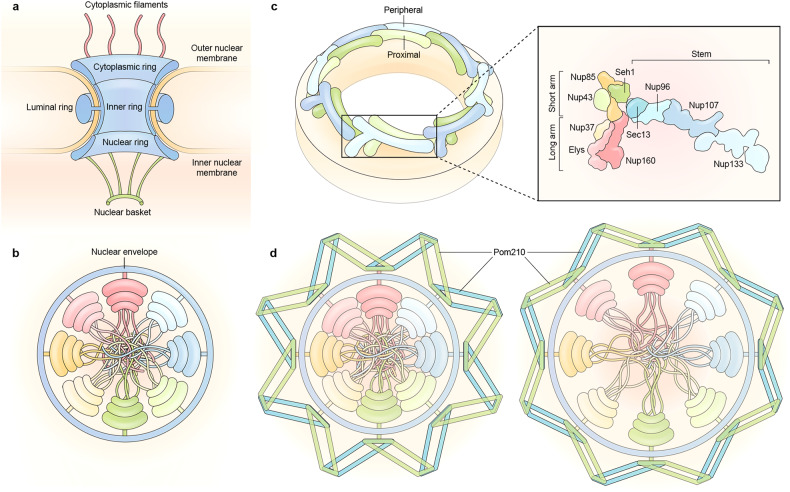
Carton representation of the nuclear pore complex. **a** The nuclear pore complex can be divided into three parts: the central core, the cytoplasmic filaments, and the nuclear basket. The central core can be further divided into four rings surrounding the central channel: the inner ring, the cytoplasmic ring, the nuclear ring, and the luminal ring. **b** Inner ring viewed in the direction of transport. The inner ring consists of eight loosely associated subunits surrounding the central channel. The central channel is filled with disordered FG repeat polypeptides that inhibit free diffusion across the nuclear envelope. **c** Architecture of the outer ring. The two outer rings (the cytoplasmic and nuclear rings) are highly similar, and only one outer ring is drawn for clarity. Each outer ring contains 16 copies of the Y complex, arranged in two concentric rings and stabilized by linker nucleoporins. The Y complex consists of 10 nucleoporins, which can be divided into three regions: the stem, the short arm, and the long arm. **d** Architecture of the luminal ring. Thirty-two copies of Pom210 connected end-to-end surround the NPC and interact with the inner ring on the other side of the nuclear envelope. The parallelogram architectural features of Pom210 allows deformation that contract (left panel) or dilate (right panel) the central channel

The NE consists of two lipid bilayer membranes—the inner and outer nuclear membranes - with NPCs embedded in NE pores where the inner and outer bilayers are curved and fused. A typical mammalian cell has approximately 2000–5000 NPCs.^29^ Each NPC can be visualized as a hollow cylinder with an outer diameter of ~1200 Å, a height of ~800 Å, and a total weight of ~120 MDa.^30,31^ The NPC can be divided into three parts: a central core that binds to the membrane and forms a diffusion barrier, eight thin filaments that bind to the central core and extend to the cytoplasm, and an additional eight thin filaments that form a basket-like structure on the nuclear side (Fig. 2a). All three parts of the NPC exhibit eightfold rotational symmetry along the channel axis, with all nucleoporins present as a multiple of eight in each NPC. The central core has an additional twofold symmetry between the cytoplasmic and nuclear halves.^30^ Therefore, each symmetric core nucleoporin (or symmetric nucleoporin) is present in at least a multiple of 16 in each NPC.^32^

Each NPC is constructed from approximately 1000 protein subunits, made up of multiple copies of approximately 34 unique nucleoporins encoded by the human genome. Approximately ten nucleoporins contain long stretches of FG repeats that are disordered and rich in FG dipeptides.^33^ These FG repeats are critical for passive diffusion barrier formation and karyopherin binding. The most prevalent domains are α-helical solenoids and β-propellers, which form the relatively rigid NPC scaffold. Nucleoporins have diverse functions, with some anchoring the NPC in the membrane (transmembrane nucleoporins), some forming the skeleton or scaffold of NPC (scaffold nucleoporins), some linking different scaffolds together (linker nucleoporins), some forming a diffusion barrier and/or interacting with different transport factors (FG nucleoporins), and some having mixed domains and functions. In this review, we introduce different nucleoporins according to their location within the NPC (Table 1). However, it should be noted that some nucleoporins are not restricted to a single location, especially those linking different parts of the NPC.

**Table 1:** The organization of nucleoporins in NPCs

		Name (copy number)	Main domain features	Functions in NPC architecture and transport
Central Core	Inner ring	Nup188 (16)	α-solenoid	Scaffold
		Nup205 (16)	α-solenoid	Scaffold
		Nup93 (32)	α-solenoid, disordered	Scaffold, linker
		Nup155 (48)	β-propeller, α-solenoid	Scaffold, linking to outer rings, membrane binding
		NDC1 (16)	Transmembrane, pore domain	Membrane anchoring, interacting with ALADIN
		ALADIN (16)	β-propeller	Membrane binding, scaffold
		Nup54 (32)	FG repeats, coiled-coil	Anchor the diffusion barrier to the NPC lumen
		Nup58 (32)	FG repeats, coiled-coil	Anchor the diffusion barrier to the NPC lumen
		Nup62 (32)	FG repeats, coiled-coil	Anchor the diffusion barrier to the NPC lumen
		Nup35 (32)	Disordered	Linker
		Nup98 (32)	FG repeats, disordered	Linker, diffusion barrier
	Cytoplasmic ring	Nup75 (16)	α-solenoid	Y short arm component, scaffold
		Nup43 (16)	β-propeller	Y short arm component, membrane binding
		Seh1 (16)	β-propeller	Y short arm component, membrane binding
		Nup160 (16)	β-propeller, α-solenoid	Y long arm component, scaffold, membrane binding
		Nup37 (16)	β-propeller	Y long arm component, membrane binding
		ELYS (8)	β-propeller, α-solenoid, disordered	Y long arm component, scaffold, membrane binding
		Sec13 (16)	β-propeller	Y stem component, membrane binding
		Nup96 (16)	α-solenoid, disordered	Y stem component, scaffold
		Nup107 (16)	α-solenoid, disordered	Y stem component, scaffold
		Nup133 (16)	β-propeller, α-solenoid	Y stem component, scaffold, membrane binding
		Nup205 (16)	α-solenoid	Linker
		Nup93 (16)	α-solenoid, disordered	Linker
	Nuclear ring	Nup75 (16)	α-solenoid	Y short arm component, scaffold
		Nup43 (16)	β-propeller	Y short arm component, membrane binding
		Seh1 (16)	β-propeller	Y short arm component, membrane binding
		Nup160 (16)	β-propeller, α-solenoid	Y long arm component, scaffold, membrane binding
		Nup37 (16)	β-propeller	Y long arm component, membrane binding
		ELYS (16)	β-propeller, α-solenoid, disordered	Y long arm component, scaffold, membrane binding
		Sec13 (16)	β-propeller	Y stem component, membrane binding
		Nup96 (16)	α-solenoid, disordered	Y stem component, scaffold
		Nup107 (16)	α-solenoid, disordered	Y stem component, scaffold
		Nup133 (16)	β-propeller, α-solenoid	Y stem component, scaffold, membrane binding
		Nup205 (8)	α-solenoid	Linker
		Nup93 (8)	α-solenoid, disordered	Linker
	Luminal ring	Pom210 (32)	Ig-like domains, transmembrane	Scaffold, membrane anchoring
		Pom121 (8)	Disordered, transmembrane	Linker, membrane anchoring
Cytoplasmic filaments	Nup358 filament	Nup358 (40)	α-solenoid, coiled-coil, disordered, RBDs	Outer ring anchoring, homopentamerization, docking platform for Ran and transport factors
	Nup214 complex	Nup214 (16)	β-propeller, coiled-coil, FG repeats	Complex with Nup62/88, transport factor binding
		Nup62 (16)	FG repeats, coiled-coil	Complex with Nup214/88, transport factor binding
		Nup88 (16)	β-propeller, coiled-coil	Membrane binding, complex with Nup214/62
		Nup98 (16)	FG repeats, disordered, GLEBS	Transport factor binding, linker, RAE1 binding
		Nup42 (16)	FG repeats, Gle1 binding motif	Transport factor binding, bind and regulate Gle1
		Gle1 (16)	Coiled-coil, α-helical	DDX19 activation
		RAE1 (48)	β-propeller	Membrane binding
		DDX19 (16)	RecA-like domain	Bind mRNA and dissociate mRNA export factors
Nuclear basket		Nup153 (32)	Disordered, ZnF, FG repeats	Basket anchoring, Ran and transport factor binding
		Nup50 (16)	FG repeats, RBD	Ran and transport factor binding
		TPR (32)	Coiled-coil, FG repeats	Scaffold, transport factor binding

*RRM* RNA recognition motif, *RBD* Ran binding domain, *GLEBS* Gle2-binding sequence

#### Symmetric core nucleoporins

The symmetric core can be further divided into four concentric rings: an inner ring which lines the central channel and forms the NPC diffusion barrier, two outer rings (nuclear ring and cytoplasmic ring) which dock the cytoplasmic filaments and the nuclear basket, and a luminal ring in the NE lumen surrounding the NPC (Fig. 2a).^34^ The inner ring and outer rings are connected by eight filaments on each side of the NPC. The filaments limit the movement of the inner ring towards outer rings but permit dilation or constriction in the NE plane. NPCs are conserved across diverse species from yeast to humans, but the degree of conservation for different parts are not the same: the inner ring, out rings, and other regions are in descending order of conservation. The inner ring thus represents the most critical part for NPC functions, especially nuclear transport.

##### Nucleoporins forming the inner ring

The inner ring is divided into eight subunits by eightfold symmetry, and each subunit is symmetrical on both the nuclear and cytoplasmic sides. When viewed from the direction of transport, each subunit resembles an eighth slice of pizza with the center portion cut away (Fig. 2b).^35^ The inner ring subunits are porous, plastic, and weakly connected to one another, allowing them to deform and change pore size in response to stimuli.^36–38^ The inner ring subunit can be further divided into three layers based on the distance to the transport axis: a middle layer of nucleoporins that form the central scaffold, an outer layer of coat nucleoporins that associate with the membrane, and an inner layer of nucleoporins that form the diffusion barriers.^39,40^

The central scaffold of each subunit is composed of two copies of Nup188, two copies of Nup205, and four copies of Nup93.^41^ These scaffold nucleoporins are mainly α-helical solenoids that intercalate extensively. The relatively rigid scaffold prevents excessive shrinkage of nuclear pores when subjected to compression force from the membrane.^42^ The coat nucleoporins include Nup155 (6 copies), NDC1 (2 copies), and ALADIN (2 copies). Nup155 contains a β-propeller domain as well as an α-helical solenoid. Four copies of Nup155 use α-helical solenoid domains to form a cushion for the central scaffold and use β-propeller domains to contact the membrane. The interaction between the inner ring and membrane is strengthened by ALADIN and NDC1. ALADIN is a β-propeller fold that interacts with the membrane and the pore domain of NDC1. NDC1 contains an additional transmembrane domain anchoring the inner ring to the NE. The two ALADIN-NDC1 heterodimers also interact with two other copies of Nup155 that contact the two outer rings.

The barrier nucleoporins, also known as the channel nucleoporin heterotrimer (CNT),^43^ include Nup54, Nup58, and Nup62 (four copies each) and are anchored by the N-terminal SLiMs of Nup93.^44^ Each of these nucleoporins contains a C-terminal coiled-coil domain bundled alongside the other coiled-coil domains of the heterotrimer, as well as an N-terminal FG repeat domain extending into the central transport channel to form the diffusion barrier. These FG repeats are depleted of charged amino acids and, at high concentrations, can self-assemble into a hydrogel-like condensate, which allows the diffusion and transport of FG-interacting karyopherins but prevents the passage of other macromolecules, biophysically similar to the NPC barrier.^45,46^ FG repeats in two disordered inner ring nucleoporins, Nup98 and Nup35 (also known as Nup53), can simultaneously bind several α-solenoid nucleoporins, which are structurally related to karyopherins, through interactions resembling those found in FG-karyopherins.^47^ In this way, these linker nucleoporins thread together all three layers, stabilize the NPC, and play a role in recruiting inner ring nucleoporins during NPC biogenesis.^39,48^

##### Nucleoporins forming the outer rings

Outer rings refer to the cytoplasmic outer ring (cytoplasmic ring) and the nuclear outer ring (nuclear ring). These two rings are largely identical, except for copy number differences of select components (ELYS, Nup205, and Nup93).^49^ Copy number differences for these proteins are also observed between species or even within a single cell, however, the functional difference remains poorly understood.^50^ Remarkably, the human outer rings contain twice as many Y-shaped structures (knowns as Y complexes or coat nucleoporin complexes, 32 vs. 16) as yeast.^51^ The outer rings bind and curve the membrane, connect the inner ring through Nup155, and form docking sites to recruit asymmetric nucleoporins (e.g., Nup358).^52^ Several asymmetric nucleoporin domains are firmly bound to outer rings and are sometimes regarded as a portion of the outer rings. For simplicity, we consider those domains to be part of the cytoplasmic filaments or nuclear baskets and will discuss asymmetric nucleoporins separately in later sections.

In each outer ring, the Y complexes are arranged head-to-tail and form two concentric rings, each containing eight copies of Y complexes (Fig. 2c). The human Y complex is composed of 10 nucleoporin proteins that form a short arm (Nup75, Nup43, and Seh1), a long arm (Nup160, Nup37, and ELYS), and a stem (Sec13, Nup96, Nup107, and Nup133), together resembling the ‘Y’ shape (Fig. 2c).^50,53^ These nucleoporins contain either α-helical solenoid domains, β-propeller domains, or both, and membrane contact is primarily mediated by the β-propeller domains at the tips of the long arm and the stem. ELYS is not considered as a component of the Y complex by some groups because it is not uniformly present in all Y complexes. In *X. laevis*, the cytoplasmic ring has eight copies of ELYS, whereas the nuclear ring contains 16 copies.^49^ The extra copies of ELYS in the nuclear ring are well-exposed, functioning in chromatin binding, decondensation and gene transcription.^50,54,55^

The cytoplasmic ring can be divided into eight identical subunits, each containing two copies of Y complexes, one proximal and one peripheral (Fig. 2c). Except for the extensive interface between the two Y complexes, two linker nucleoporins, Nup205 and Nup93, connect and stabilize the two Y complexes. In addition, these two nucleoporins mediate inter-subunit interactions in a head-to-tail fashion, strengthening the outer ring scaffold. In *Xenopus laevis*, the cytoplasmic ring contains two copies of Nup205 and Nup93, while the nuclear ring subunit contains only one copy each of Nup205 and Nup93.^49,56^ Unlike the inner ring, the outer rings have extensive intersubunit interactions and rigid linkers, thus not allowing intersubunit movements. Stable outer rings are capable of restricting the inner ring movement through the Nup155 filament.^38^

##### Nucleoporins forming the luminal ring

The luminal ring (also known as the membrane ring) is within the perinuclear lumen of the NE and equatorially encircles the NPC.^57^ The luminal ring may sense membrane tension, set the NPC dilation limit, and buffer collisions with adjacent NPCs.^34,37,58^ The luminal ring appears as eight arches connected end to end and can be conceptualized as 16 parallelograms joined on their short sides (Fig. 2d).^58,59^ Since the luminal ring is connected to the inner ring via NDC1, the deformation of parallelograms can contract or dilate the associated inner ring. The deformation of parallelograms is likely passive, allowing the NPC to adapt to membrane tension and transport demands. The luminal ring contains Pom121 and Pom210 in vertebrates, both possessing a single-pass transmembrane region.^42,57^ Pom210, which contains 16 immunoglobulin-like domains, is responsible for the CryoEM density of the luminal ring, since each Pom121 contributes merely ~30 residues to the luminal ring.^58^ The pore side of Pom121 is largely unstructured and directly binds the β-propeller domains of Nup155 (the inter-ring filament) and Nup160 (Y complex component), thereby anchoring the cytoplasmic ring to the membrane.^60,61^ Whether and how Pom121 directly bind Pom210 are currently unclear.

#### The cytoplasmic filament nucleoporins

The cytoplasmic filaments are anchored to the cytoplasmic outer ring and possess long, flexible filamentous extensions into the cytoplasm. The exact architectural details of these extensions are not fully understood due to their conformational heterogeneity. These filaments are composed of less conserved accessory nucleoporins, being cell-type specific and modifiable by cellular processes.^62,63^ Nevertheless, cytoplasmic filaments play a crucial role in the recruitment of transport factors and the final step of protein and mRNA export.^64^ Nup214, Nup358, Nup98, and Nup42 are the main contributors to cytoplasmic FG repeats.^33^

Most of the molecular mass of the cytoplasmic filament is contributed by Nup358, which is large in size (358 kD) and high in copy number (five copies per filament).^52^ Five Nup358 molecules form a homopentameric complex using the coiled-coil domains and assemble onto the stems of two Y complexes using the N-terminal α-helical solenoid domains.^52^ Nup358 assembly in turn can stabilize the Y complex rings.^65^ The remaining domains of the five Nup358 molecules are entangled and flexibly extend into the cytoplasm, forming the observed 50 nm filamentous structures.^66^ The extended region of Nup358 contains four dispersed RanBP1-like Ran binding domains, a tandem array of eight zinc-finger RanGDP-binding domains, a binding site for the SUMO E2 ligase Ubc9 and RanGAP1, many FG repeats, and a catalytically active cyclophilin domain.^67^ These domains are involved in RanGTP hydrolysis, RanGDP recycling, and karyopherin docking.^68–71^

Alongside Nup358, the cytoplasmic ring is decorated with 16 copies of Nup214 complexes.^50^ This complex is constructed by eight nucleoporins including Nup214, Nup62, Nup88, Nup98, Nup42, Gle1, RAE1, and the ATP-dependent DEAD-box RNA helicase DDX19, although some of these proteins are not constitutively associated with NPCs.^51^ An earlier study showed that Nup358 assembly is dependent on the Nup214 complex, but the reverse is not true.^72^ Nup214, Nup88, and Nup62 uses the coiled-coil domains to form a heterotrimeric complex similar to the one observed in CNT. This complex is anchor to the short arm of the Y complexes and to the membrane, forming a multivalent interaction hub.^50,52^ Two other subcomplexes, Nup98/RAE1 and Nup42/Gle1/DDX19, are recruited to the vicinity using long linkers. The Nup214 complex thus localize critical factors to remove mRNA from its export factors in the final step of mRNA export.^73^ Unlike Nup358-mediated protein export termination, this process is independent of Ran and occurs closer to the central channel, but the biological significance is unclear.

#### Nuclear basket nucleoporins

In humans, the nuclear basket is made up of three nucleoporins: Nup50, Nup153, and Tpr. Tpr is the major structural component of the basket, as it has a large coiled-coil domain which allows for homo-oligomerization.^74^ Prior studies have demonstrated that Nup153 is responsible for tethering Nup50 to the nuclear pore and post-mitotic recruiting of Tpr to NPC, but not for stabilizing Tpr that is already anchored within the NPC.^75–77^ Unlike the cytoplasmic face, the nuclear face of NPC had minimal electron density beyond the symmetric core nucleoporins, indicating that the basket is anchored by short linear motifs.^49,50,78^ In agreement with this, depletion of multiple Y complex components, e.g., Nup75 (a Y short arm component) and Nup133 (a Y complex stem component), perturbed nuclear basket formation.^59,79^ Amphipathic helices from Nup1 (Nup153 orthologue) in yeast can induce membrane curvature and stabilize the nuclear ring.^75,80^ Nup50 and Nup153 forms a cargo disassembly station for nuclear import due to containing high affinity FG repeats interaction sites for importins.^78^ Besides nuclear transport, the nuclear basket is critical for cellular processes such as mRNA production and quality control, chromosome organization, and DNA damage repair,^75,81,82^ but how and why these processes occur at this location are largely unknown.

### Karyopherins responsible for ferrying cargo across the nuclear pore complex

Karyopherins are molecules that ferry cargoes across NPCs either into or out of the karyo-compartment (the nucleus). These proteins are conserved from yeast to humans and are important in many cellular processes.^83^ Typical karyopherins are divided into three groups: importins, which import cargoes into the nucleus; exportins, which export cargoes to the cytoplasm; and biportins, which transport cargoes in either direction.^4^ These karyopherins rely on the GTPase RanGTP for cargo binding and dissociation.^84^ They are large in size (~ 1000 residues), forming alpha-helical solenoid structures.^20^ Generally, the highly acidic concave surface is used for interactions with RanGTP and cargo, and the convex surface presents hydrophobic pockets to interact with the FG repeats of the NPC. There are several small size transport factors that are not known as karyopherins, but similar to karyopherins, they can transport cargo through NPCs.^85^ On the other hand, a group of transport adaptor molecules (alpha karyopherins) are known as karyopherins, but they cannot independently transport cargo.^86^ In this section, each of these factors is explained in terms of the cargoes it recognizes, the mode in which cargoes are recognized, the cellular pathways in which it may specialize, and the associated diseases (Table 2).

**Table 2:** Members of broad sense karyopherins

	Name (abbrv)	Synonyms	Cargo examples	Binding motif/domain	Cargo clustering
Importin	Importin β1 (Impβ1/Impβ)	KPNB1, Kapβ1,	Many cargoes through the adaptors Impα1 and Snurportin, SREBP-2, NFκB, TFEB, PD-L1	A basic helix by IBB, globular domains	DNA synthesis, DNA repair, gene expression regulation
	Transportin 1 (TNPO1/TRN1)	Kapβ2	hnRNP A1, TDP-43, FUS, β-catenin, BAP1	Linear PY-NLS, RGG domain, poly PR peptide	RNA processing, nuclear division, tRNA ligases
	Transportin 2 (TNPO2/TRN2)	IPO3, Imp3	hnRNP A1, TDP-43, FUS	Linear PY-NLS,	RNA processing, DNA repair, HMG proteins
	Transportin 3 (TNPO3)	TrnSR2, TNPO-SR	ASF/SF2, CPSF6, CIRBP, HIV virus,	Linear motif R(pS/D/E)R(pS/D/E)	Transcription elongation, mRNA splicing and export
	Importin 4 (IPO4/Imp4)	RanBP4	Histones H3 and H4, Vitamin D Receptor, CEBPD	LPPRS(G/P)P linear motifs, histone H3-H4 globular domain	DNA metabolic processes, chromosome organization
	Importin 5 (IPO5/Imp5)	RanBP5, KPNB3	Ribosomal proteins, histone H3, influenza A virus RNA polymerase subunit PB1, DSCAM	Linear motif KP(K/Y)LV	Ribosomal biosynthesis
	RanBP6		STAT3		
	Importin 7 (IPO7/Imp7)	RanBP7	Histone H1, HIV reverse transcription complex (RTC), Smad1, Yap1, DNA plasmids	Disordered histone tail	mRNA splicing factors, snRNPs, HMG proteins
	Importin 8 (IPO8/Imp8)		Smads, eIF4E, microRNAs		mRNA splicing factors, ribosomal proteins
	Importin 9 (IPO9/Imp9)	RanBP9	ARID3A, PFKP, histones H2A and H2B.	Histone H2A-H2B globular domain	Ribosomal proteins or mRNA splicing factors.
	Importin 11 (IPO11/Imp11)		BZW1/2, β-catenin, PTEN.		Developmental processes
Exportin	Exportin 1 (XPO1, Exp1)	CRM1	p53, FOXO, Survivin, TFEB, cGAS.	Linear NES motif Φ-X_1–3_-Φ-X_1–3_-Φ-X_1–3_-Φ.	A broad spectrum of cargoes
	Exportin 2 (XPO2)	CAS, Cse1, Cse1L	Impα1–7	Globular domain	Impα only
	Exportin t (XPOT)		Mature tRNA	tRNA stem and two ends	Mature tRNA only
	Exportin 5 (XPO5)		Pre-miRNA, tRNA, hairpin RNA, RNA coexported proteins	The stem and 3’ overhang	Some double-stranded RNAs
	Exportin 6 (XPO6)		Actin		
Biportin	Importin 13 (IPO13)		Import GR, Mago-Y14, Ubc9, PDCD5, export eIF1A	Globular domains of Mago-Y14, Ubc9, and eIF1A	Chromatin modification, chromatin remodeling, and transcription.
	Exportin 4 (XPO4)		Sox2, SRY, PKM2, Smad, eIF5A, a subset of circRNAs in metazoans	Globular domains of eIF5A	RNAP II elongation factors, mRNA processing factors.
	Exportin 7 (XPO7)		RhoGAP1, 14-3-3sigma, p65	Basic folded domains	Diverse functions.
	RanBP17				
Smaill size	NTF2		Ran, CapG	GDP-bound Ran	Import of Ran and piggyback proteins
	NXF1 family	TAP	mRNA, piRNA		Nuclear export of different RNAs
	Hikeshi		Hsp70	Full length protein	Only Hsp70 import
Adaptor	Importin α family	KPNA1/Impα5, KPNA2/Impα1, KPNA3/Impα4, KPNA4/Impα3, KPNA6/Impα7, KPNA7/Impα8,	NFκB, STATs, eVP24, PB2	Classical NLS containing one or two stretch(s) of polyK/R sequences	Broad range of cargoes
	Snurportin	Snurportin 1	m3G-capped U snRNPs		m3G-capped U snRNPs

The name and synonyms include only those that have been used in recent years

#### The importins that import cargoes into the nucleus

**Fig. 3:**
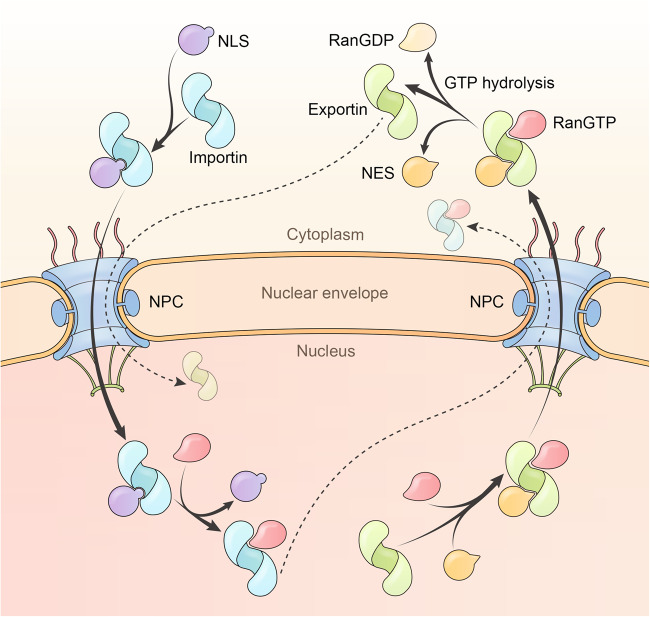
Model of protein nuclear import and export. Imported cargoes containing nuclear localization signals (NLSs) form complexes with importins in the cytoplasm, enter the nucleus through the NPC, and are dissociated from the importins with the aid of RanGTP. Nuclear export of cargoes starts with the formation of trimeric complexes consisting of exportin, nuclear export signal (NES)-containing cargo, and RanGTP. The trimeric complex transits through the NPC and is dissembled in the cytoplasm upon the hydrolysis of RanGTP. Certain species of RNA utilize protein adaptors to cross NPCs

Humans possess ten verified importins.^87^ The function of RanBP6 is unclear, but it is classified as an importin because it has high sequence homology (80% identity) to Importin 5. Importins bind to cargoes in the cytoplasm and release cargoes within the nucleus upon encountering the GTP-bound form of the GTPase Ran (Fig. 3). Generally, cargo binding and RanGTP binding are mutually exclusive, but RanGTP binds with a greater affinity and is thus able to dissociate cargoes.^4^ A proteomics study demonstrated that each importin recognizes a set of cargoes, although many of these interactions require further verification.^88^ Importins recognize cargoes in diverse ways, but all rely on positively charged amino acids in cargoes.^4^ This may explain why many cargoes are able to enter the nucleus using multiple importins.^89,90^

##### Importin β1

The most widely studied importin is Importin β1 (Impβ1, also known as Importin β, karyopherin β1, or by its gene name KPNB1). Impβ1 acts as a transporter in classical nuclear import, wherein it recruits the adaptor protein Importin α (Impα, containing 7 isoforms) that directly binds to the cargo protein. The N-terminal Importin Beta Binding Domain (IBB) of Impα is basic and forms an α helix upon binding to Impβ1.^91^ Likewise, Impβ1 imports m3G-caped U snRNA by binding to the IBB of the adaptor snurportin.^92^ The use of adaptors enhances the diversity of cargoes recognized by Impβ1 and allows for fine regulation of nuclear import.^93^ Impβ1 may also directly recognize and import cargo without adaptors, for example, binding globular domains of the cholesterol metabolism transcription factor SREBP-2 to mediate its nuclear import.^94^ As a major import receptor, Impβ1 imports many cargoes, including the NF-κB subunit p65, autophagy transcription factor TFEB, and programmed cell death ligand 1 PD-L1.^95–97^ Most Impβ1 cargoes play a role in DNA synthesis and repair, as well as gene expression regulation.^88^ Impβ1 abnormalities are present in several diseases, such as upregulation in cancers and downregulation in neurodegenerative diseases.^83,98^

##### Transportin 1 and Transportin 2

Transportin 1 (TNPO1, also known as karyopherin β2) and Transportin 2 (TNPO2, also known as Importin 3) are highly homologous (85% sequence identity) and well-characterized importins that bind the PY (proline-tyrosine) nuclear localization signal (NLS) of cargo proteins.^99^ A typical PY NLS is disordered and contains two patches, an N-terminal positive/basic patch and a C-terminal [**+**]-X_2–5_-P-ϕ motif ([**+**], positively charged residue; ϕ, any hydrophobic residues including Y).^99^ In addition to the PY NLS, these importins bind arginine-glycine-glycine (RGG) domains in RNA binding proteins such as hnRNP A1, FUS, and the proline-arginine (PG) poly-dipeptides from C9orf72.^100–103^ As such, impairment of TNPO1 and TNPO2 causes those proteins to aggregate and condense in neuron cells, contributing to neurodegenerative diseases.^83^ Furthermore, TNPO1 imports the Wnt signaling effector β-catenin as well as the tumor suppressor BAP1, and many viruses exploit TNPO1 for nuclear entry and replication.^104–107^ Proteome analysis indicated that proteins related to nuclear division and tRNA ligases are preferentially cargoes of TNPO1, while proteins related to DNA repair and HMG proteins are preferentially imported by TNPO2.^88^ Interestingly, TNPO2 enhances export of a large proportion of mRNAs through the formation of a complex with RanGTP and the mRNA export factor NXF1,^108^ suggesting that it may be appropriate to classify TNPO2 as a biportin.

##### Transportin 3

Transportin 3 (TNPO3, also known as Transportin-SR or Transportin-SR2) specifically binds cargoes containing an arginine/serine (RS) domain.^109^ Counter intuitively, serine phosphorylation of TNPO3 cargoes reduces net positive charges but enhances TNPO3 binding.^110^ Structural analysis has demonstrated that TNPO3 uses a unique arginine-rich helix for interaction with phosphorylated serine residues, in addition to the common acidic patches that interact with positively charged residues in importin cargoes.^111^ Examples of TNPO3 cargoes include the alternative splicing factor/splicing factor 2 (ASF/SF2), cold-inducible RNA-binding protein (CIRBP), and polyadenylation specificity factor 6 (CPSF6).^101,111^ Many TNPO3 cargoes are RS-rich splicing factors.^88^ The HIV virus utilizes TNPO3 to facilitate its nuclear import and replication, and a natural TNPO3 mutation that causes limb girdle muscular dystrophy also provides strong resistance against HIV-1 infection.^112,113^ Refer to the Supplemental File for reviews on other importins.

#### The exportins that export cargoes to the cytoplasm

The human genome encodes five exportins to conduct the export of cellular proteins and RNAs. Unlike importins, exportins display low affinities to either RanGTP or the cargo, typically in the micromolar range. However, exportins can cooperatively bind cargoes and RanGTP and form nanomolar affinity complexes in the nucleus. After translocating through NPCs to the cytoplasm, the complex is disassembled via RanGTP hydrolysis.^114^ Each exportin recognizes cargo by a different mechanism, and there is no common rule for cargo recognition.^115^

##### Exportin 1

Exportin 1 (XPO1, Exp1, also known as chromosomal region maintenance protein 1, CRM1) is the best characterized exportin.^115^ There are approximately 200 validated XPO1 protein cargoes, including p53, FOXO, Survivin, TFEB, and the cyclic GMP-AMP synthase cGAS.^116–118^ XPO1 cargoes are often involved in translation, cytoplasmic mRNA metabolism, vesicle coat complexes, and centrosome proteins.^119,120^. XPO1 interacts with leucine-rich nuclear export signals (NES), which are typically made up of four large hydrophobic residues separated by 1–3 linker residues (conforming to a Φ-X_1–3_-Φ-X_2–3_-Φ-X_1–3_-Φ motif).^121^ These hydrophobic residues are arranged linearly and bind in a long groove on the convex side of XPO1.^122^ The groove opens and closes dynamically, and RanGTP binding to the concave side stabilizes the open groove conformation.^123^ On the other hand, cargo binding displaces a loop on the concave side (H9 loop) and prepares XPO1 for RanGTP binding. Utilizing different protein adaptors, XPO1 can also export a variety of RNA molecules.^124,125^ XPO1 is frequently overexpressed in cancers and impairs the function of many tumor suppressors by exporting them to the cytoplasm.^126^

##### Exportin 2

Exportin 2 (XPO2, cellular apoptosis susceptibility, CAS, or chromosome segregation 1-like, Cse1, Cse1L) is a dedicated nuclear export factor for the classical nuclear import adaptor Impα, which is unable to traverse NPCs alone.^127^ By wrapping around RanGTP and Impα and folding the IBB in the NLS binding sites of Impα, XPO2 ensures cargo dissociation from Impα before export.^128^ XPO2 depletion alters the localization of multiple silencing factors and reactivates many repressed genes, due to its indispensable role in classical nuclear import.^129^ As Impβ1, XPO2 is overexpressed in many cancers.^130,131^

##### Exportin 5

Exportin 5 (XPO5) exports pre-miRNA, and this step is necessary for proper miRNA maturation.^132,133^ The crystal structure illustrates that Exp-5:RanGTP recognizes the 2-nucleotide 3’ overhang structure and the double-stranded stem of pre-miRNA.^134^ Likely through the same RNA interface, XPO5 exports tRNA and other double-stranded RNA molecules, as well as co-exports proteins bound to these RNAs.^135–138^ Impaired miRNA maturation due to XPO5 dysregulation such as genetic mutation and phosphorylation-mediated inhibition has been observed in several cancers.^139,140^ However, XPO5 was reported to be expressed in colorectal cancer that promotes the expression of oncogenic miRNA, but how this is selective for oncogenic miRNA but not tumor-suppressive miRNA is not clear.^140^ Exportin 6 and Exportin t are reviewed in the Supplementary File.

#### Bidirectional transporters

Biportins can function as importins to import cargoes or as exportins to export cargoes. There are three verified biportins in humans, and RanBP17 is classified as a biportin due to its high sequence homology to the biportin Exportin 7. The use of dedicated importins and exportins may allow for more diverse cargo recognition modes and more specific pathway control. On the other hand, using biportins in transport is likely more economical than using importins and exportins separately.

##### Importin 13

Importin 13 (IPO13) is a well-characterized biportin which imports glucocorticoid receptor GR, the exon junction complex components Mago-Y14, the E2 SUMO-conjugating enzyme Ubc9, programmed cell death 5 PDCD5, while exporting translation initiation factor 1 A eIF1A.^141–143^ A proteomic study demonstrated that IPO13 binds to many cargoes functioning in chromatin modification, chromatin remodeling, and transcription.^88,144^ Crystal structures of IPO13 in complex with Mago-Y14, Ubc9, and eIF1A have illustrated the mechanism by which this importin uses different surfaces to interact with different cargoes and how it plastically changes conformation upon binding to different cargoes.^142,145^ IPO13 overexpression plays a role in several cancers, and loss-of-function mutations cause defects in eye morphogenesis,^146–148^ but which cargo(es) mediate these pathological consequences are unknown.

##### Exportin 4

Exportin 4 (XPO4) mediates nuclear import of transcription factors Sox2 and SRY, the glycolytic enzyme PKM2, as well as mediates nuclear export of Smad proteins, the hypusine-containing translation factor eIF5A, and interestingly, a subset of circRNAs.^149–152^ Many XPO4-imported cargoes identified by mass spectrometry are RNAP II elongation factors and mRNA processing factors.^88^ The export cargo eIF5A is bound to the convex and concave surface of XPO4, with the hypusine bound in an acidic pocket.^152^ It is unclear how XPO4 recognizes other cargoes, but its plasticity may play a role in binding to different cargoes. Reduced expression of XPO4 due to copy number variation sustains nuclear Smad levels and TGFβ signaling, thereby enhancing the severity of fibrosis in patients with metabolic-associated fatty liver disease.^153^

##### Exportin 7 and RanBP17

Exportin 7 (XPO7) was initially identified as an exportin for RhoGAP1 and 14-3-3sigma.^154^ It was then demonstrated that XPO7 could also recognize positively charged folded domains and mediate the nuclear import of NFκB/p65.^155^ A recent proteomic study showed that XPO7 may import and export hundreds of cargoes with diverse structures and functions.^156^ How XPO7 recognize cargoes has not been reported. Depletion of XPO7 correlates with poor overall survival in several cancer types due to lack of oncogene-induced senescence caused by insufficient nuclear localization of p21 transcription factor TCF3.^157^ RanBP17 is 67% identical to XPO7 but little is known about this protein.

#### Smaller size transport factors

There are a few smaller-sized transport factors that do not form α-solenoid. However, they function as karyopherins and are capable of recognizing cargoes and translocating through NPCs.^158^ Unlike typical karyopherins, they are very specific in cargo recognition, do not rely on the RanGTP for cargo binding and dissociation, and contain fewer FG pockets.^159^

##### Nuclear Transport Factor 2

One ‘small karyopherin’ is Nuclear Transport Factor 2 (NTF2), which contains only 127 amino acids. NTF2 is a dedicated RanGDP nuclear import factor that recycles inactive RanGDP to the nucleus.^160^ NTF2 forms a homodimer and uses a distinct hydrophobic cavity for recognition of RanGDP.^161,162^ Two identical FxFG binding sites within the dimer are used for FG binding and NPC translocation.^158^ How NTF2 is dissociated from RanGDP in the nucleus is unclear, but NTF2 inhibits the guanine nucleotide exchange activity of RCC1 on Ran.^163^ Nuclear translocation of Ran may simultaneously import ankyrin repeat proteins and the filamentous actin capping protein CapG via a piggyback mechanism.^162,164,165^

##### NXF1 family

The nuclear RNA export factor 1 (NXF1, also known as TAP) family of proteins possesses an NTF2 domain capable of FG repeat binding and interacts with NTF2-like export factor 1 (NXT1) to form a heterodimer reminiscent of the NTF2 homodimer.^166,167^ This heterodimer binds to FG repeats but not RanGDP.^166^ NXF1 facilitates mRNA nuclear export since it also contains several other domains that interact with RNA and other mRNA processing factors, such as the TREX complex.^168^ Unspliced RNAs are generally not exported, but type-D retroviruses use a ∼130 nucleotide RNA called the constitutive transport element (CTE) to bind NXF1-NXT1 without protein adaptors (e.g., TREX) to export their unspliced genomic RNA.^169^ Structural analysis shows that this CTE-RNA forms a symmetrical stem-loop motif that binds to a symmetrical site formed by two copies of NXF1-NXT1 dimers.^167^ In humans, NXF1 is a major mRNA export factor, but there are a few other less-understood NXF family export factors, such as NXF2 and NXF3.^170,171^ NXF2 appears to be a tissue-specific mRNA export factor.^171^ Interestingly, NXF3 lacks FG binding pockets and instead relies on binding to XPO1 to translocate through NPCs, illustrating the diversity of RNA export.^125,172^

##### Hikeshi

The heat shock nuclear import factor Hikeshi contains 197 a.a. and is structurally unrelated to NTF2. Under conditions of heat shock, importins are globally downregulated and Hikeshi mediates nuclear import of molecular chaperone Hsp70 to counteract heat-shock damage and increase cell viability.^173^ Hikeshi contains an FG-binding N-terminal domain (NTD) and a C-terminal dimerization domain, and forms an asymmetric dimer that recognizes the full-length ATP-bound Hsp70.^174^ Interestingly, an loop in NTD contains a FG motif that can dock into its own FG pocket, thereby autoinhibits its interaction with FG nucleoporins and nuclear import function. How this autoinhibition is lifted under heat shock, how Hikeshi recognizes Hsp70, and whether Hsp70 is exported by Hikeshi after completing its nuclear function are unclear.^175^

#### The transport adaptor molecules

The transport adaptor itself does not have NPC translocation capabilities; however, it can bind karyopherin and cargo at the same time, thereby facilitating cargo transportation. They play important roles in nuclear transport, and in fact, alpha karyopherins are the first ‘karyopherins’ identified.^176^ Any protein that contains an NES or NLS and forms a tight complex with another protein/RNA is a potential nuclear transport adaptor. Because the list of adaptors is very long, except for the few examples shown above, two classes of well-studied adaptors with broad utility are reviewed here.

##### Importin α family

The importin α (Impα, or karyopherin α) family of adaptors functions in classical nuclear import, and it recognizes classical NLS signals. A classical NLS contains one or two stretches of polyK/R (2-4) sequences which bind to one or two acidic patches in the concave surface of Importin α.^177^ Impα contains an N-terminal Importin beta binding (IBB) domain that directly binds to Impβ1.^178^ This IBB can also bind to its own NLS binding sites, playing an autoinhibitory role so that cargo binding only occurs in the presence of Impβ1.^179^ In humans, there are seven Impα family members (Impα1 - Impα7) that are ~ 50%–80% identical in sequence and completely identical in the NLS interaction surface.^180^ These members are not entirely redundant, as they differ in cargo specificity and tissue- or developmental-stage-specific functions.^181–184^ Impα binds to a broad range of cargoes, including NFκB, STAT transcription factors, Ebola virus VP24 protein (eVP24), and influenza Polymerase PB2, thereby often involved in different cancers and viral infections.^185–188^

##### Snurportin

Snurportin (also known as Snurportin 1, SNUPN) is the nuclear import adaptor for m3G-capped U snRNPs, which participate in pre-mRNA splicing.^92^ Similar to Impα, Snurportin uses an IBB domain to interact with Impβ1.^189^ SNUPN contains an NES and is recycled to the cytoplasm via XPO1.^190^ Structural analysis has revealed that SNUPN binds to XPO1 in a manner incompatible with snRNP binding, thereby ensuring cargo unloading prior to nuclear export.^191^

### Ran system proteins determining transport direction and speed

**Fig. 4:**
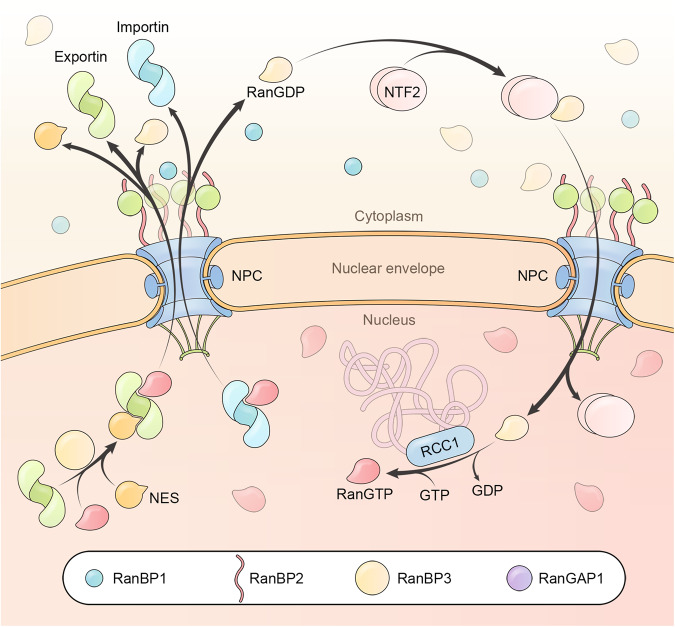
The RanGTP system and regulatory proteins. Ran is predominantly GTP-bound in the nucleus and GDP-bound in the cytoplasm. RanGTP is exported to the cytoplasm in complexes with karyopherins (either importins or export-cargo-bound exportins). In the cytoplasm, RanBP1 or RanBP2 (Nup358) promotes the dissociation of RanGTP from karyopherin complexes, allowing RanGAP1-mediated GTP hydrolysis. RanGDP is recycled back to the nucleus by NTF2, dissociated from NTF2, and reloaded with GTP by chromatin-bound RCC1 (GEF). RanBP3 enhances the recruitment of RanGTP to exportins

The transport directionality of importins, exportins, and biportins relies on an elaborate RanGTP system.^5^ This system generates the RanGTP gradient, strictly partitioning RanGTP in the nucleus and RanGDP in the cytoplasm.^192^ This RanGTP gradient is maintained by the nuclear-specific distribution of Ran guanine nucleotide exchange factor (GEF) RCC1 and the cytoplasm-specific localization of the GTPase-activating protein (GAP) RanGAP1 (Fig. 4).^193^ RanGDP, which is continuously generated throughout transport, is recycled to the nucleus by the aforementioned NTF2. In addition to these essential factors, four Ran binding proteins (RanBP1, RanBP2, RanBP3, and Nup50) regulate the interaction between RanGTP and karyopherins, accelerating transport speed.^194^

#### Ran

The Ras-related nuclear protein Ran contains a typical small GTPase domain and a C-terminal flexible tail that interacts with Ran-binding domains (RBD).^195^ Ran is active when it is GTP-bound and inactive when it is GDP-bound. RanGTP typically binds importins with nanomolar to picomolar affinities, and its binding either directly clashes with the cargo or induces an importin allosteric change to induce cargo dissociation.^196,197^ The binding affinity between an exportin and its cargo is usually higher than micromolar; however, RanGTP and the cargo cooperatively bind to the exportin at an affinity of tens to hundreds of nanomolar.^198^ In the cytoplasm, RanGTP in the export complex (either RanGTP-importin or RanGTP-exportin-cargo) is hydrolyzed to RanGDP through the cooperative action of RanGAP1 and RanBP1/2.^67^ In mitotic cells, RanGTP production is localized to chromosomes to promote local spindle assembly and at a later stage, local NE and NPC assembly.^199–201^

#### RCC1

Regulator of chromosome condensation (RCC1) contains an NLS that mediates its nuclear import and binds chromatin in the nucleus.^202^ RCC1 directly binds nucleosomal DNA via its N-terminal tail and a DNA binding loop, and it binds nucleosomal histones via a switchback loop.^203,204^ It collapses the P-loop of Ran to release bound nucleotides, and increases guanine nucleotide dissociation by over five orders of magnitude.^205,206^ GTP/GDP exchange catalyzed by RCC1 is indiscriminate, but due to the greater abundance of GTP compared to GDP in cells, nuclear Ran is eventually charged with GTP.^205^ Because NTF2 inhibits RCC1-mediated nucleotide exchange, an unknown ATP-dependent factor dissociates RanGDP from NTF2 to allow for RCC1-catalyzed guanine nucleotide exchange.^163,207^ Chromatin-bound RCC1 is responsible for local production of RanGTP in mitotic cells.

#### RanGAP1

RanGAP1 (RanGAP) is a cytoplasm-localized Ran-specific GAP recruited to the cytoplasmic filament protein Nup358 when SUMOylated.^69,208,209^ RanGAP1 does not use an arginine finger but positions Ran’s catalytic glutamine in the active conformation to trigger hydrolysis.^210^ RanGAP1 is anchored to the kinetochore and mediates chromatid segregation during mitosis, and depletion of RanGAP1 drives chromosome instability and tumorigenesis.^211,212^

#### RanBP1, RanBP2, RanBP3, and Nup50

RanGTP is tightly wrapped within karyopherins and is inaccessible to RanGAP1 when nuclear export complexes (RanGTP-importin or RanGTP-exportin-cargo) enter the cytoplasm.^213,214^ RanBP1 is a coactivator of RanGTP hydrolysis that increases the rate of RanGAP1-mediated RanGTP hydrolysis by an order of magnitude.^215^ This is achieved through its Ran-binding domain (RBD), which tightly binds to RanGTP and increases the rate of karyopherin-RanGTP dissociation.^127,216^ RanBP1 contains an NES and is located exclusively in the cytoplasm.^217^ RanBP2 (also known as Nup358) contains four RBDs functioning similarly to RanBP1, namely, in dissociating RanGTP from karyopherins and allowing RanGAP1-mediated GTP hydrolysis.^218^ In contrast, RanBP3 is a nuclear-localized RBD-containing protein that promotes nuclear export cargo assembly.^219^ RanBP3 contains several FG sequences that can form high-affinity anchors with exportins and an RBD domain, facilitating recruitment of RanGTP to exportins.^220^ This lowers the entropic barrier for RanGTP loading, as exportins typically have low affinity for RanGTP.^221,222^ Basket-localized Nup50 contains a high-affinity importin-binding FG domain and a C-terminal RBD that can recruit RanGTP to accelerate cargo dissociation from importins.^223,224^ These domain features of Nup50 enable it to increase the rate of nuclear import complex disassembly and, ultimately, nuclear import.

RanBP2, SUMOylated RanGAP1, and Ubc9 together form the NPC-localized SUMO E3 ligase, thus potentially linking SUMOylation and nuclear transport.^225–227^ SUMOylation of sites within or adjacent to the NES or NLS can disrupt karyopherin binding or alter the binding partner of modified proteins to render them inaccessible to karyopherins, thereby altering protein localization.^228,229^ SUMOylation of a protein may also enhance its nuclear import or export, but the mechanisms are largely unknown.^230^ On the other hand, nuclear transport also regulates protein SUMOylation. The nuclear import of many proteins, such as Sp100 (a component of the PML nucleosome), is critical for their SUMOylation.^231,232^ Although these proteins may undergo SUMOylation during nuclear entry, their SUMOylation may also occur inside the nucleus by other SUMO E3 ligases. In fact, most SUMO-modifying enzymes and SUMOylated proteins, including many kinetochore proteins, are found in the nucleus.^233,234^ For example, the kinase Aurora B, a key regulator of mitosis, is SUMOylated at the centromere in early mitosis by SUMO ligases including the RanBP2 complex.^235^ Interestingly, NPC also binds to deSUMOylase. The major de-SOMOylating enzyme SENP2 localizes to NPCs by binding to Nup153 and is critical for the de-SUMOylation of ribosomal precursors and their subsequent nuclear export.^236,237^ Removal of the highly hydrophilic SUMO groups may reduce energy required to penetrate the hydrophobic NPC barrier, which is especially important for translocation of large cargoes such as ribosomal precursors. Exported proteins could theoretically be SUMOylated by the RanBP2 complex, but reports on this are limited.^67^ Among many SUMO-regulated processes, gene expression, DNA damage response, and immune response can occur in the vicinity of NPCs, and future discoveries on how NPC-mediated SUMOylation participates in these processes to impact diseases such as tumors and infections are anticipated.^238,239^ It remains largely unclear what determines whether a translocating cargo is SUMOylated and how SUMOylation and nuclear transport cooperate in specific pathways.

### Translocating across the NPC barrier

Nucleoporins, karyopherins, and Ran system proteins work together to transport cargo through the NPC. Each NPC can transport cargo at a staggering rate of ~ 1000 molecules per second, especially considering that it simultaneously prevents non-specific passive diffusion.^240^ The passive diffusion size limit is reported to be 40 kD, but few macromolecules employ passive diffusion to cross NPC due to low efficiency.^241^ Although much is known about the individual NTPs, we remain uncertain how NPC simultaneously achieve such a high level of transport efficiency and selectivity. Both the barrier and its interaction with karyopherin are highly dynamic and complex, making them difficult to study with most existing techniques.^242^ The variety of different models that have been proposed highlights our current lack of consensus in this regard.^243–247^

The nature of the barrier is highly debated. For example, whether the barrier is cohesive or non-cohesive, or in simpler terms, whether the barrier is formed of highly condensed “hydrogels” or more dynamic and loosely packed “polymer brushes”.^248,249^ GLFG repeats containing nucleoporins such as Nup98 are highly cohesive and form hydrogels in vitro at physiological concentration, but charged FG nucleoporins are less cohesive and do not naturally form hydrogels.^250,251^ The hydrogels formed in vitro exhibits many characteristics similar to the NPC barrier.^252^ It is not difficult to imagine that by anchoring in the relatively rigid NPC scaffold, the GLFG repeats are locally enriched,^253^ thereby forming a hydrogel barrier in NPC.^244,254^ However, this raises the question of whether and how karyopherins can rapidly melt and thus pass through cross-linked gels as rapidly as observed. Furthermore, high-speed atomic force microscopy revealed that the center of the barriers was entangled but ‘did not condense into a tightly cross-linked network’.^255^ Another model proposed that the highly dynamic FG repeats prevent the passage of non-interacting macromolecules by means of entropic exclusion, i.e., FG repeats exclude passive diffusion by forming a non-cohesive and highly entropic ‘virtual gate’.^22,256^ Invasion of inert macromolecules limits the entropy of the FG nucleoporins, thus being energetically unfavorable. Regardless of the debates, it is now known that the FG domains account for only ~ ¼ of the molecular mass of the NPC lumen, with the other ¾ being karyopherins and the cargoes they carry.^257^ A number of studies have highlighted a ‘karyopherin-centric’ model whereby karyopherins are integral constituents of the barrier and are critical for preventing NPC leakage.^258–260^ This model could complement both the hydrogel model (to reduce cohesiveness) and the virtual gating model (to outcompete non-specific diffusion).^259,261^ Although NPCs are somewhat heterogeneous in composition, it is unlikely that different gating mechanisms exist in different NPCs, but more likely that they coexist in all NPCs.^262^

**Fig. 5:**
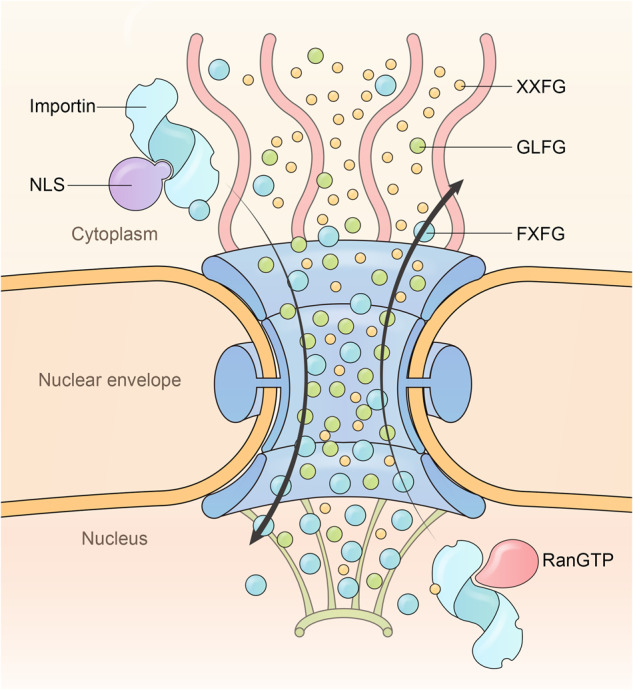
Model of uni-directional translocation within NPCs. In NPC, XXFG (small ball), GLFG (medium ball), and FXFG (large ball) repeats are not uniformly distributed but exist in concentration gradients. NLS binding to importin in the cytoplasm rearranges importin HEAT repeats to generate large pockets with high affinity for FXFG repeats, thereby moving importin and the bound cargo toward the nucleus with the aid of the FG gradient. Nuclear RanGTP binding renders importin surface pockets small and selective for XXFG repeats, driving the export of the complex. Exportin can also take advantage of this FG concentration gradient. Concentration gradients of different FG repeats provides a traction force and restrains directionality, thereby contributing to the high transport efficiency of NPCs. This model also explains the biological significance of the cytoplasmic filaments and the nuclear basket in nuclear transport (see text)

Another controversy concerns the process of translocation, i.e. how karyopherins (with or without cargo) translocate from one side of NPC to the other.^263^ One of the earlier models proposed that certain FG nucleoporins bind karyopherins on one side of the NPC, escort them across the barrier, and release them on the other side.^224,264^ However, data generated later showed that the interaction between FG repeats and karyopherins is rather dynamic: FG pockets rapidly binds, dissociates, and rebinds other FGs in vicinity.^265^ Thus, rather than remaining tightly bound to FGs of one nucleoporin throughout transport, it is more plausible that karyopherins rapidly “slide” on the FG repeats of different nucleoporins to move forward.^266^ The ‘Brownian motion’ model suggest that the translocation process in the barrier is energy-independent and directionless.^267–269^ However, this model does not account for two important facts: (1) the distribution of different types of FGs, i.e., XXFG, GLFG, and FXFG, is asymmetrical in NPCs,^270,271^ and (2) cargo-loaded karyopherins always have a stronger affinity for the FG type on their destination side.^272–275^ Further, uncontrolled movement of karyopherins may cause traffic congestion and reduce transport efficiency, especially for large cargoes. Therefore, an ‘affinity gradient’ model demands that, besides RanGTP control of transport direction outside the barrier, the trafficking inside the barrier is constrained to a single direction, with the asymmetrically distributed FG types establishing an affinity gradient for karyopherins and luring it towards the high affinity end.^273,274^ Here, we further add that different FG types can be abstracted as hydrophobic balls of different sizes, with FXFG, GLFG, and XXFG representing large (2 F), medium (1.5 F), and small (1 F) balls, respectively (two Fs in FXFG or LF in GLFG are held together when inserted into FG pockets, as illustrated by different crystal structures, Fig. 5). RanGTP and cargo binding regulate the FG pocket size of karyopherin, shaping it selective for certain size balls, as observed in the ‘reversible collapse’ model where Importin β1 but not Importin β1-RanGTP can bind and collapse FXFG containing Nup153.^249^ Take importin as an example, after cargo binding, its FG pocket enlarges to bind 2 F balls and move along the 2 F gradient to the basket side. In the nucleus, RanGTP binding reshapes the importin FG pocket to select for 1 F balls and drive the importin to the cytoplasmic side. Free importins tend to stay in one compartment (cytoplasm for most importins) since it is energetically unfavorable to move against the affinity gradient. This prevents energy wasting, since GTP is consumed during transport (via RanGTP hydrolysis) even when there is no cargo. Furthermore, this model could explain why more karyopherins are required for large cargoes to cross the NPC:^276^ more karyopherins provide more energy (moving down the affinity gradient yields energy) or traction force to overcome the energy required for penetration of large cargoes. The affinity gradient can provide each karyopherin with energy equal to that generated by RanGTP hydrolysis in one round of import and export, if not considering any energy loss. Along this thread, the cytoplasmic filament and nuclear basket can use the affinity gradient to select export complexes and import complexes, and only at these exposed locations, these complexes are terminated by RanGTP hydrolysis and RanGTP binding, respectively. Without these cytoplasmic and nuclear extensions, these import and export complexes may spend a longer time in the transport channel where RanGTP and RanGAP are excluded, and ultimately ruduce transport efficiency. This model could better explain the observed high efficiency of NPC transport.

## Nuclear transport-independent functions of nuclear transport proteins

In interphase cells, NTPs play an important role in mediating the nuclear import and export of macromolecules. In mitotic cells, these proteins continue to regulate aspects of mitosis through fundamental principles of nuclear transport.^277^ Many NTPs act as molecular chaperones for highly basic cargoes to prevent aggregation and cellular degradation or form biomolecular condensates through phase separation.^278^ Some NTPs also interact extensively with chromatin, regulating its structure and transcription.^279^ Readers are redirected to these works for other atypical NTP functions such as cilia transport and nuclear sizing.^280–283^

### Mitosis

In mitotic cells, the RanGTP system signals the location of chromosomes as a global positioning system (GPS). The RanGTP system cooperates with karyopherins to modulate key mitotic factors that are usually cargoes of karyopherins. Many nucleoporins are also critical players in mitosis and are highly involved in different stages of mitosis. These NTPs orchestrate many aspects of mitosis, and their defects may lead to genetic instability and tumorigenesis through different mechanisms. Although less studied, meiosis is also regulated by NTPs due to its similarity to mitosis.^284,285^

#### The role of RanGTP, Impβ1, and Impα1 in mitosis

**Fig. 6:**
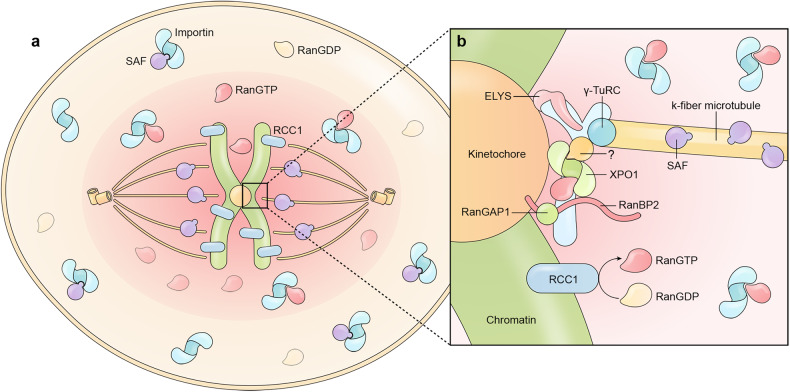
Role of nuclear transport proteins in mitotic spindle assembly and chromatid segregation. **a** Chromosome-bound RCC1 catalyzes the production of RanGTP around chromosomes. RanGTP near the chromosomes dissociates spindle assembly factors (SAFs) from bound importins, and the released SAFs promote spindle assembly. In the cortical region of the cell, spindle assembly is inhibited by excess importins. **b** The Y complex nucleoporins are critical for the recruitment of γ-TuRC, which induces k-fiber formation (kinetochore-initiated spindle microtubules). XPO1 is recruited to the Y complex and strengthens the connection between the k-fiber and the kinetochore. RanBP2 and RanGAP1 are recruited by XPO1 and mediate chromatid segregation at anaphase

In mitotic cells, RCC1 is constantly bound to chromosomes, continues to generate RanGTP surrounding the chromosome and promotes local mitotic spindle assembly and functioning.^286^ Defects in RCC1 localization or function perturb the RanGTP gradient, resulting in chromosomal misalignment, abnormal spindle pole number, abnormal chromosome segregation, and genome instability.^287^ RanBP1 can form a tight complex with RCC1/RanGTP and inhibit RCC1 function, thereby regulate spatial distribution and magnitude of mitotic Ran-GTP production at different stages.^288^ Many spindle assembly factors (SAFs), including NuMA, HURP, TPX2, and APC, are classical nuclear import cargoes, and a high concentration of RanGTP in the vicinity of the chromosome releases SAFs from Impβ1 and Impα1 (Fig. 6a).^277^ These released SAFs participate in microtubule nucleation, growth, stability, and organization.^289^ In the cortical region, Impβ1 and Impα1 inhibit the mitotic function of SAFs by binding to the NLS of these SAFs. NLS binding by Impβ1 and Impα1 often sterically masks the functioning region of an SAF, e.g., the microtubule-binding region of NuMA.^290^ This intricate system prevents spindle assembly at nonchromosomal locations. RanGTP regulation of spindle assembly is not a switch but rather a gradient from the chromosome to the cell cortex where importins and SAF activities are gradually tuned.^291^

In addition to spindle assembly, the RanGTP-Impα1/β1 system regulates many other events in mitosis. For example, Impα directly competes with p115, a vesicle-tethering factor, for the interaction with the Golgi matrix protein GM130, inhibits p115/GM130-mediated vesicle fusion, and promotes Golgi disassembly in the early stages of mitosis.^292^ During anaphase, the TPX2 NLS is phosphorylated, and the bound importin α and β1 are dissociated, allowing Eg5 recruitment to promote centrosome separation.^293^ The formation of NE in late mitosis requires Lamin B-coated NE precursor vesicle-vesicle fusion at the vicinity of chromatin, a process possibly induced by local dissociation of Impβ1 from Lamin B receptor, which then simultaneously binds chromatin and NE vesicles.^294^ Likewise, nuclear pore assembly is also regulated by RanGTP and classical nuclear import karyopherins.^295,296^ These studies collectively highlight a model in which RanGTP regulates the activity of many mitotic factors through Impα1/β1 at different mitotic stages to ensure proper chromosome segregation.

#### The role of other karyopherins in mitosis

In addition to Impβ1/Impα1, TNPO1, TNPO2, and potentially other importins, are involved in mitosis.^277^ For example, TNPO1 is known to inhibit recruitment of the Y complex to kinetochores and suppress mitotic spindle assembly, a process that is counteracted by RanGTP.^297^ Furthermore, inhibiting TNPO1 induces the formation of microtubule asters in the mitotic cytosol, while cells depleted in TNPO1 display defects in spindle and cytokinesis.^298^ Other importins are rarely reported in mitosis regulation, but their importance should not be neglected because they can also bind many mitotic factors.^88^

As anticipated, exportins are involved in mitosis. In particular, exportin XPO1 is recruited to kinetochores via RanGTP- and NES-binding and is required for stabilizing the connection between kinetochores and k-fibers (kinetochore-initiated spindle microtubules).^299^ XPO1 is also present at centrosomes, recruiting pericentrin and γ-tubulin ring complex (γ-TuRC) to nucleate spindles from the centrosome.^300^ In addition, centrosome-localized XPO1 may also recruit other NES proteins such as NPM, p53, BRCA1, and cyclin B, to ensure the mitotic fidelity and prevent genomic instability.^301^ For example, XPO1-RanGTP controls the spatial/temporal recruitment of NES-containing NPM to prevent centrosome reduplication.^302^ Generation of the microtubule organizing center (MTOC) at the NPC in yeast requires Nup159 (human Nup214), XPO1, RanGTP, and the MTOC protein Mto1, where XPO1 simultaneously binds the Nup159 FG domain and the Mto1 NES to link them together.^300^ The E3 ligase Nup358/RanGAP1/Ubc9, which is recruited to kinetochores by XPO1, SUMOylates and recruits TopoIIσ to decatenate sister centromeres prior to anaphase onset.^299,303^ Nup358 haploid mice develop cells with anaphase bridges and severe aneuploidy, and are highly susceptible to tumor formation.^257^

#### The role of nucleoporins in mitosis

As shown in Fig. 6b, some of the dismantled NPC parts, especially the Y complex, are recruited to kinetochores and centrosomes, where they continue to function during mitosis.^304^ The recruitment of the Y complex to the kinetochore occurs through its component ELYS, and this process is inhibited by Impβ1 and TNPO1 that compete with kinetochores for ELYS binding.^297,305^ The kinetochore Y complex recruits γ-TuRC which induces k-fiber formation.^285,306^ The presence of the Y complex at kinetochores is required for the recruitment of XPO1 as well as chromosome passenger complex (CPC) proteins, a critical factor in chromosome alignment and segregation.^307,308^

Moreover, the proper expression levels of Nup98/Nup88/RAE1/Tpr/Nup153 are critical for spindle polarity, preventing aneuploidy and tumorigenesis.^309^ Mechanistically, Nup98 and RAE1 form a complex with the anaphase-promoting complex/cyclosome (APC/C) to inhibit premature progression into anaphase through inhibition of APC E3 ligase activity.^310^ Sequestration of Nup98/RAE1, by RAE1/Nup98 haploinsufficiency or overexpression of Nup88, can activate APC/C and induce degradation of the mitotic kinase PKL1, disrupt normal centrosome separation, and lead to aneuploidy.^311^ The spindle assembly checkpoint (SAC) proteins Mad1 and Mad2 associate with Tpr at NPC in interphase cells and are recruited to kinetochores in mitotic cells without Tpr, signaling to inhibit APC function until all kinetochores are attached to spindles.^312^ Cyclin B1-CDK1 is targeted to NPC at early mitosis and mediates Tpr phosphorylation to release Mad1 so that it can be recruited to kinetochore.^313–315^ In conclusion, studies have shown that NTS proteins play an indispensable role in mitosis, and that their dysfunction can distort mitosis and lead to genomic instability and cancers by different mechanisms. More studies are needed to reveal the complex spatiotemporal interactions and regulation mechanisms of NTPs in mitosis.

#### Disassembly and reassembly of NPC during mitosis

During mitotic entry, NPCs break down into subcomplexes within approximately 5 min and disperse to different regions of the cell.^316^ Phosphorylation of several nucleoporins is a decisive event for NPC disassembly and subsequent entry into mitosis.^317,318^ The responsible kinases include cyclin-dependent kinase 1 (CDK1), polo-like kinase 1 (PLK1), and NIMA-associated kinases (NEKs) NEK6/7.^317,318^ The CNT complex can recruit PLK1 to NPCs during mitosis in *C. elegans*.^319^ These kinases primarily hyperphosphorylate two nucleoporins, Nup98 and Nup35.^318^ Hyperphosphorylation of more than 15 sites in the Nup98 C-terminal domain dissociates Nup98 from NPCs and is the rate-limiting step in mitotic NPC disassembly.^317^ Nup98 and Nup53 are linker nucleoporins linking different NPC subcomplexes, and hyperphosphorylation of the intermolecular interaction sites disrupts their linker function, leading to their dissociation from NPC, dissociation of threaded subcomplexes such as the CNT complex, and exposure of more nucleoporins to be phosphorylated and disassembled.^38,318^ Y complexes are not further dissembled, but released into the mitotic cytoplasm, or recruited to kinetochores and assist in spindle assembly, or retained in the membrane with transmembrane nucleoporins to serve as templates for later reassembly.^320^ These studies highlight the role of hyperphosphorylation and inactivation of key linker nucleoporins in NPC disassembly.

It takes ~10 min to reassemble the NPC after anaphase onset.^321^ To start, the Y complex binds to chromatin via the C-terminal disordered region of ELYS.^322^ ELYS also recruits the phosphatase PP1, which dephosphorylates phosphorylated nucleoporins to allow their assembly.^323,324^ Chromatin-bound RCC1 and a high concentration of RanGTP in vicinity are critical in this process, since RanGTP relieves the inhibition of several importins on chromatin-Y complex interaction.^277^ Nup50 can bind and stimulate the activity of RCC1 and is also critical for NPC assembly.^223^ Mitotic NPC assembly and NE assembly are tightly coupled, possibly through the transmembrane nucleoporins.^325^ Membrane-embedded Pom121 can interact with the Y complex, allowing the nuclear membrane to form around newly formed (partial) NPCs.^61,326,327^ Another transmembrane nucleoporin, NDC1 is also critical for anchoring NPCs to membranes, since it interacts with (dephosphorylated) Nup35 which stabilize the inner ring subcomplexes.^328^ The recruitment of Nup98 to the inner ring and the Y complex may further stabilize the NPC scaffold.^48^ EM studies show that the cytoplasmic ring is assembled after the nuclear ring and inner ring.^329^ The mitotic reassembly of NPC described above is largely the reverse process of disassembly, but studies suggest that there may exist multiple reassembly pathways, e.g., cytoplasmic assembly of NPC precursors.^330–332^ More studies are needed to determine the proportion and detailed steps of different assembly pathways.

### Regulation of biomolecular condensates

Owing to their acidic surface properties, karyopherins, especially importins, interact with many highly basic cargoes (HBCs).^333^ This interaction not only plays a role in the nuclear import of these HBCs but also prevents their binding to other cellular targets and sometimes their cellular degradation.^278^ A special group of HBCs is the RNA binding proteins (RBPs), including FUS, hnRNP A1, and TDP-43. These proteins contain intrinsically disordered regions that can phase separate within the cytoplasm to form membraneless liquid droplets or β-amyloid-like solid fibers.^278^ The chaperone activity of importins also disaggregates already oligomerized RBPs and may be exploited to halt or reverse neurodegeneration. In contrast, many FG nucleoporins can phase separate to form hydrogel-like permeable barriers or aggregate with other cellular condensates, playing key roles in physiological or pathological processes.

#### Ability to function as a molecular chaperone

Many highly abundant HBCs, such as histones and ribosomal proteins, readily aggregate with cytoplasmic polyanions such as RNAs.^25^ As early as 2002, Jakel *et al*. demonstrated that several importins, such as IPO4, IPO5, IPO7, IPO9, and Impβ1, can serve as chaperones for these HBCs. The chaperone activity of these importins requires their large acidic surfaces, which shield the basic patches in HBCs and thereby prevent the ionic aggregation of HBCs with cellular polyanions. This is conceptually similar to canonical chaperons that prevent hydrophobic aggregation of proteins with large hydrophobic surfaces. This chaperone activity not only prevents aggregation but also protects the HBCs from proteasome-mediated degradation, since aggregated proteins are prone to aggregation.^334,335^ Recent studies have confirmed and expanded upon this role.^336,337^ It has been shown that the disassembly of the IPO9-H2A-H2B complex requires the presence of DNA in addition to nuclear RanGTP.^338^ This stricter dissociation mechanism may also allow for the storage of unused histones.^338^ The chaperone function is not limited to importins, as it has shown that XPO4 can bind to the export cargo eIF5A and inhibit its undesired interactions before entering the cytoplasm.^152^

#### Ability to disaggregate RBPs

**Fig. 7:**
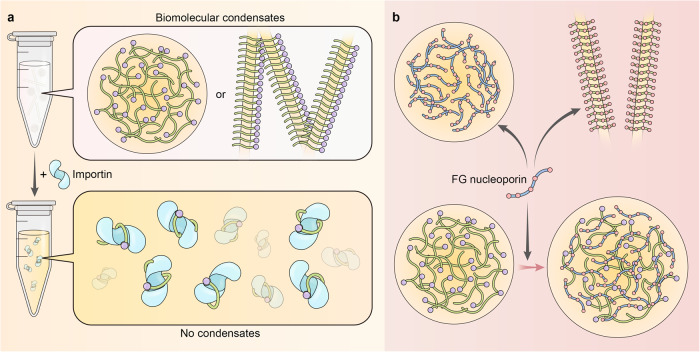
Role of nuclear transport proteins in regulating biomolecular condensates. **a** Importins can act as molecular chaperons for highly positively charged cargoes, such as many RNA binding proteins. The interaction between importin and cargo inhibits cargo aggregation and disaggregates biomolecular condensates (including fibers) formed by cargo. Purple dots represent nuclear localization signal of cargo proteins. **b** Many FG nucleoporins can form biomolecular condensates either on their own and/or coaggregate with other biomolecular condensates, such as TDP-43 droplets and stress granules. Red dots represent FG dipeptides

Some importin-chaperoned HBCs are the neurodegenerative disease-associated RBPs, including FUS, TAF15, hnRNP A1/A2, and TDP-43.^278^ These RBPs are typically larger and contain RNA recognition motif (RRM) domains, intrinsically disordered low complexity (LC) regions, and arginine-glycine-glycine rich (RGG) domains. These domains contain weak and multivalent interaction sites, predisposing these RBPs to undergo phase separation with or without RNA. Phase-separated RBPs can further form amyloid fibers under certain conditions.^339^ Importins, in contrast, inhibit their self-association and even dissolve aggregated RBPs (Fig. 7a).^340^ For example, TNPO1 inhibits and reverses fibrils formed by PY-NLS-containing FUS, TAF15, hnRNPA1, and hnRNPA2. Similarly, Impα and Impβ1 prevent and reverse TDP-43 fibrillation.^341,342^

The mechanism of importin-mediated RBP disaggregation is starting to be unveiled. First, an intact NLS in the cargo is required for the chaperone activity of importins. Equimolar importin are often required to fully dissolve the preformed hydrogels or fibrils.^342^ Second, binding to NLS alone is not sufficient to inhibit aggregation, since an antibody against FUS NLS did not inhibit FUS self-association.^343^ Similarly, XPO1 did not inhibit FUS aggregation when the NLS of FUS was replaced with an NES.^344^ This suggests that TNPO1 forms additional contacts with FUS in addition to its NLS interactions. However, these interactions are very weak, dynamic, and difficult to visualize using typical structural biology approaches.^344^ These interactions likely involve the acidic surfaces of importins and positively charged residues in the RRM/RGG domains of cargoes, as well as the FG pockets of importins and the FG dipeptides (or FG-like hydrophobic residues) of cargoes.^278^ For example, FUS contains two FG dipeptides and 19 FG-like YG dipeptides, and TDP-43 contains 8 FG dipeptides. Therefore, through NLS binding, importins may reduce the phase separation ability of RBPs by sequestering key elements that drive phase separation.^345^

#### Ability to form biomolecular condensates

FG domains are intrinsically disordered and contain weak multivalent interaction sites, such as positively charged residues and F/Y residues that can form π-cation interactions, and thus capable of phase separation like other intrinsically disordered domains. FG nucleoporins can be found in various cellular condensates, including amyloid-like fibers (Fig. 7b).^346,347^ Within the nuclear pore, the concentrated FG domains may aggregate into a condensed phase to form the selectively permeable barrier, although this is under debate.^348,349^ Karyopherins, which can form multivalent interaction with FG domain through FG pockets, coexist in these FG condensates and can fortify the NPC barrier to prevent NPC leakage. Additionally, many FG nucleoporins can phase-separate with other aggregation-prone proteins.^248,350,351^ Direct interaction between Nup98 and Tau is observed to promote phase separation of each other in vitro.^352,353^ Furthermore, fragments of Nup98 and Nup214, when fused with other DNA binding domains, can phase separate at chromosomal regions, a process that induces chromosomal looping and regulates transcription.^354,355^

### Transcription regulation

As nuclear transport impacts the localization of different transcription factors, nuclear transport is naturally a critical step in transcription regulation.^356^ However, mounting evidence suggests that many nucleoporins can directly regulate transcription, independent of their function in nuclear transport.^279,357^ These nucleoporins can interact with transcription factors at promoters and enhancers.^358^ The end result may be either transcriptional activation or inhibition, and this regulation does not necessarily occur at the NPC locus.^359^ These activities are cell-type specific, and their dysregulation may drive the initiation and progression of different tumors.^360,361^

#### Transcription activation

More transcriptionally active genes are localized to NPCs from yeast to humans.^362^ Recruitment of active genes to NPCs may facilitate transcription factor binding immediately following nuclear import and coordinate transcription with subsequent nuclear export.^363,364^ Multiple studies have shown that nucleoporins can induce promoter-enhancer interactions, activating transcription.^362,365^ These nucleoporins are mainly from the nuclear outer ring and the nuclear basket.^366^ For example, Nup93 and Nup153 bind transcription factor-rich super-enhancers and drive the expression of key genes that specify cell identity.^367^ The nucleoporin Seh1 promotes transcription of proteins essential for oligodendrocyte differentiation through assembly of an Olig2-dependent transcription complex.^368^

Nucleoporins have been found to play a role in transcriptional activation not only at the nuclear pore complex but also in the nucleoplasm.^359^ Insides the nucleoplasm of Drosophila, several nucleoporins (Nup98, Nup62, and Nup50) interacts with development and cell cycle genes and activates their transcription.^369^ Similarly, Nup88 also binds to silent loci off-pore, and these nucleoporin-binding loci are often distinct from those NE contact sites.^370^ Nup98 can promote transcription by stimulating the ATPase activity of the DExH/D-box helicase DHX9.^371^ In leukemia, Nup98 is frequently fused to other DNA-binding homeodomain proteins, such as HOXA9, leading to the expression of oncogenes to drive leukemogenesis.^372^ The phase separation property of fusion nucleoporins seems critical for the transcription regulation activity. Interestingly, their condensation to chromatin depends on the chromatin-bound XPO1 that has formed a complex with RanGTP and a chromatin-bound NES-containing protein.^373^ Inhibition of XPO1 by leptomycin B disrupts the interaction of these nucleoporins with chromatin and reverses transcriptional activation mediated by these nucleoporins.^373^

#### Transcription repression

Less frequently transcribed heterochromatin is usually enriched at the nuclear periphery.^374^ Nup93 is associated with polycomb-silenced genes and physically interacts with a group of polycomb proteins, and polycomb repressive complexes containing Nup93 are more stable and localized to the nuclear periphery.^375^ Therefore, Nup93 may repress transcription by promoting heterochromatin formation.^375^ Similarly, Nup153 associates with the transcriptional start site of developmental genes and recruits polycomb-repressive complex 1, maintaining stem cell pluripotency in mammalian cells.^376^ Nup88 binds specifically to silenced genes; however, the regulatory mechanism is unclear.^370^

Genes near telomeres are less frequently transcribed due to the ‘positional effect’.^377^ Telomeres are localized at the nuclear periphery and bind silencing factors, such as Sir2, Sir3, and Sir4.^378^ In yeast, the Y complex component Nup170 (human Nup155), as well as the nuclear basket components Mlp1/2 (human Tpr), are critical for maintenance of the correct localization of telomeres.^379,380^ Furthermore, these nucleoporins can recruit silencing factors to telomeres.^381^ Depletion of these nucleoporins results in defective telomere silencing.^382^

#### Bimodal regulation

Actively transcribing genes are usually grouped into distinct topologically associated domains (TADs) with boundaries on both sides of the domain that insulate transcription within a TAD.^383^ The nuclear basket protein Nup153 interacts with key boundary proteins CTCF and cohesion to stabilize TADs.^365^ Therefore, Nup153 depletion leads to improper TAD boundaries as well as differential gene expression.^365^ Another study demonstrated that promoter binding by Nup153 increased gene expression, while transcriptional end site binding reduced gene expression.^384^ While it is conclusive that many nucleoporins can regulate transcription, whether phase-separation is involved in all these interactions and whether other NTPs regulate the process are largely unclear.

## Diseases involving defects in nuclear transport proteins

Due to their high functional importance and relatively low gene redundancy, many NTPs are key players in different diseases. In particular, cancer cells often upregulate the expression of many karyopherins to alter the localization of cargoes or promote oncogenic transcription by creating nucleoporin fusion proteins.^4,385,386^ Defects in different NTPs downregulate nuclear transport and improperly localize key RNA-binding proteins such as TDP-43 in different neurodegenerative diseases.^83^ Many viruses exploit nuclear transport machinery to complete their life cycle in hosts and/or suppress host immune responses through impairment of nuclear transport.^387,388^ Inhibitors targeting various NTPs are being actively developed and clinically tested in relevant diseases.

### Cancer

In the mitosis section, we showed that NTP dysfunction can result in improper mitosis, genetic instability and cancers. Cancer cells also frequently overexpress karyopherins or employ mutations to manipulate the localization of key proteins and RNAs to promote proliferation and evade tumor suppression. The most prominent example is overexpression or mutation of XPO1, which localizes many tumor suppressors to the cytoplasm to render them inactive. In addition, the transcriptional regulation function of several NTPs is also implicated in cancers. In leukemia patients, fragments of Nup98 and Nup214 are frequently fused to other proteins, resulting in fusion proteins that promote oncogenic transcription.

#### Overexpression of NTPs in cancer

**Fig. 8:**
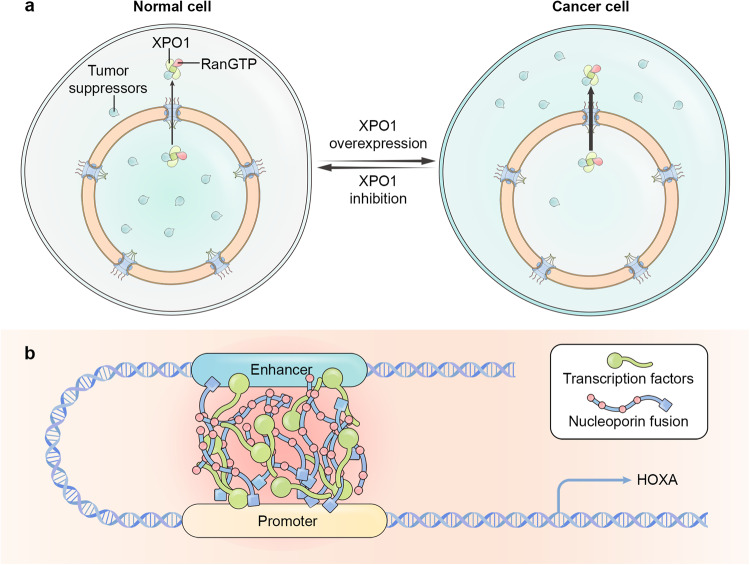
Role of nuclear transport proteins in cancer. **a** Overexpression of XPO1 leads to mislocalization and subsequent inactivation of many tumor suppressors, such as P53. Inhibition of XPO1, for example, by the FDA-approved drug selinexor, correctly repositions the tumor suppressors in the nucleus and inhibits cancer cell growth. **b** In several hematopoietic cancers, nucleoporin fusion proteins cocondensate with other transcription factors around chromatin, induce aberrant chromatin looping and activate HOXA cluster oncogenes

Cancer cells often exhibit increased nuclear translocation velocity and capacity in response to faster signaling and metabolic stress, and many NTP proteins are overexpressed in cancer.^98,389^ XPO1 overexpression in many types of cancer correlates with disease severity and prognostic outcome in various studies.^390^ Mislocalization and inactivation of tumor suppressor proteins, such as P53, P21, and Rb, in the cytoplasm have been linked to XPO1 overexpression (Fig. 8a).^391^ Since overexpression of XPO1 is required to sustain multiple hallmark features of cancer,^392^ genetic or pharmacological inhibition of XPO1 is effective in a broad spectrum of cancer cells.^131^ XPO1 frequently mediates drug resistance, and XPO1 inhibitors were reported to enhance the efficacy of many clinically used drugs.^115,393^ However, the first-generation XPO1 inhibitor leptomycin B failed clinical trials due to high toxicity.^394^ Leptomycin B covalently binds to XPO1 and permanently inhibits its nuclear export function, but XPO1 is essential for the survival of all eukaryotic cells.^16,395^

Several reversible second-generation XPO1 inhibitors have been subsequently developed with significantly reduced toxicity.^396–398^ Among them, selinexor (KPT-330) was approved by the FDA in 2019 for the treatment of relapsed and refractory diffuse large B-cell lymphoma as well as multiple myeloma.^115^ Dozens of selinexor clinical trials are underway, either as a single agent or in combination with other therapies (Table 3).^399–401^ Eltanexor (KPT-8602), a next-generation inhibitor that is fast reversible and less permeable to the blood-brain barrier, is also being investigated in several clinical trials.^402^ These studies clearly demonstrate the efficacy of XPO1 inhibitors in patients with advanced and refractory human cancers, especially hematological cancers, either alone or in combination with pre-existing therapies. The adverse effects are mostly gastrointestinal and hematological, such as nausea, vomiting, anemia, and thrombocytopenia. In elderly patients with acute myeloid leukemia, reduced survival with selinexor was observed, but the reasons were unclear.^403^ See these reviews for earlier clinical trials.^115,126,131^

**Table 3:** Recently completed clinical trials involving XPO1 inhibitors

Studies	Treatments	Efficacy (total number of patients)	High grade (>3) AEs	References
Advanced soft tissue sarcomas, phase I	Selinexor 60 or 80 mg weekly plus 75 mg/m2 doxorubicin	Partial response 21% and stable disease 63% (24)	Neutropenia (56%), febrile neutropenia (28%) and anemia (24%)	^494^
Advanced or metastatic malignancies, phase I	Selinexor 40 mg/m2 twice a week given 2 out of 3 weeks	Complete response 2.7% (74)	Hyponatremia (23%), fatigue (8%), vomiting (5%), thrombocytopenia (5%), and anemia (2%)	^495^
Relapsed or refractory multiple myeloma, phase I	Eltanexor (KPT-8602) 5, 10, 20, 30, and 40 mg, given once daily for 5 days per week or 60 mg at days 1, 3, 5 of each week	ORR 33% (39)	Thrombocytopenia (54%), neutropenia (33%), and anemia (18%)	^496^
Refractory/relapsed adult acute myeloid leukemia, phase I	Selinexor 60 mg (3 patients), 80 mg (3 patients), and 100 mg (7 patients) weekly	Complete remission 42% (12)	Non-hematologic AEs in 78.6% of patients	^497^
Patients with chronic lymphocytic leukemia and non-Hodgkin lymphoma, phase I	Weekly oral selinexor and daily oral ibrutinib	ORR 32% (34)	Thrombocytopenia (24%), anemia (18%), and neutropenia (12%)	^498^
Recurrent metastatic solid tumors, phase Ib	Selinexor (60 mg or 80 mg twice weekly orally) and weekly paclitaxel (80 mg IV 2 week on, 1 week off)	ORR 17% (24)	Neutropenia (46%), anemia (31%), and nausea (21%)	^499^
Advanced or metastatic solid tumors, phase 1b	Selinexor 40 or 60 mg combined with different standard chemotherapy	Disease control rate 14% (19)	Neutropenia (42%), leukopenia (26%), and hyponatremia (21%)	^500^
Previously treated multiple myeloma, phase I/II	Selinexor (100 mg once per week), bortezomib (1·3 mg/m2 once per week), and dexamethasone (20 mg twice per week)	Median PFS 13.93 months (195)	Thrombocytopenia (39%), fatigue (13%), anemia (16%), and pneumonia (11%)	^501^
Hypomethylating agents refractory myelodysplastic syndromes, phase I/II	Eltanexor 20 mg or 10 mg, days 1–5 each week	ORR 53% (15)	Infrequent	^502^
Relapsed/refractory multiple myeloma, phase II	Selinexor 80 mg combined with dexamethasone 20 mg	ORR 29.3% (82)	Anemia (57.3%), thrombocytopenia (51.2%), lymphopenia (42.7%), neutropenia (40.2%), hyponatremia (29.3%), and lung infection (26.8%).	^503^
Elderly patients with acute myeloid leukemia and high risk myelodysplastic syndrome, phase II	Standard chemotherapy with or without oral selinexor 60 mg twice weekly	Event-free survival without selinexor 45% (51) versus with selinexor 26% (51)	With selinexor, cardiac AEs (11%), gastrointestinal AEs (43%), infectious AEs (57%), metabolic and nutritional disorders AEs (46%)	^403^
Heavily pre-treated Chinese patients with relapsed/refractory multiple myeloma, phase II	Oral selinexor 80 mg combined with dexamethasone 20 mg, twice a week	ORR 29.3% (82)	Anemia (57.3%), thrombocytopenia (51.2%), lymphopenia (42.7%), neutropenia (40.2%), hyponatremia (29.3%), and lung infection (26.8%)	^503^
Recurrent glioblastoma, phase II	Selinexor 50 mg/m2 or 60 mg twice weekly, or 80 mg once weekly	Six-month PFS 12.6% (68)	Serious AEs in 34% patients; 1.3% fatal	^400^
Refractory diffuse large B-cell lymphoma, phase IIb	Selinexor, 60 mg and 100 mg, twice weekly	ORR 29.1% (134)	Thrombocytopenia, lymphopenia, neutropenia, anemia, or hyponatremia in ≥15%	^504^
Advanced, refractory dedifferentiated liposarcoma, phase II/III	Selinexor 60 mg twice weekly	PFS medians 2.8 months (188)	Nausea (80.7%), decreased appetite (60.4%), and fatigue (51.3%)	^505^
Previously treated multiple myeloma, phase III	Oral selinexor (100 mg) and subcutaneous bortezomib (1.3 mg) once weekly and dexamethasone 40 mg per week	PFS median 11.76 for 2–3 prior lines (108)	Manageable and generally reversible AEs	^506^

*PFS* progression-free survival, *ORR* objective response rate, *AE* adverse effect

Canonical nuclear import factors Impβ1 and Impα1 are overexpressed in multiple cancers, albeit less frequently than XPO1.^87^ Overexpression of these proteins may lead to nuclear entry of many oncogenic transcription factors (such as PDL1 and β-catenin) to promote tumorigenesis.^98,404^ The overexpression of other Impα isoforms and other karyopherins, such as XPO5, XPO6, and Imp8, has been reported in a few specific cancer types.^140,184,405–407^ In addition to karyopherins, other NTPs, such as Ran, Nup93, and POM121, have also been reported to be overexpressed in cancers, playing a role in cancer initiation and/or progression.^356,408,409^ Mechanistically, overexpression of nucleoporins and Ran is unlikely to cause cancer via promoting nuclear transport, but rather through their other functions such as mitotic and transcriptional regulation functions.

#### Mutations that change protein localization

Mutation also plays a role in altering cellular localization of key proteins in cancers. Notably, the XPO1 E571K mutation is present in a quarter of patients with Hodgkin lymphoma and primary mediastinal B-cell lymphoma.^410^ Moreover, E571K accelerated leukemogenesis in a mouse model of chronic lymphocytic leukemia.^389^ This mutation altered XPO1 localization and the interactome of XPO1 in B-cell lymphoma.^411^ Structurally, E571 is located proximal to the NES groove, and the E571K mutation can increase the affinity for XPO1 cargoes that have more acidic residues in the NES sequence.^412^

Many studies have identified pathogenic mutations occurring within cargoes rather than karyopherins.^87^ For example, the tumor potential of cyclin D1 depends on its nuclear retention.^413^ T286 mutations, which specifically disrupts cyclin D1 phosphorylation and XPO1-mediated nuclear export, have been found in primary esophageal carcinoma samples.^413^ Moreover, nucleophosmin (NPM), which is localized in the nucleus of normal cells, is cytoplasmic in approximately one-third of acute myeloid leukemia samples and plays a key role in leukemogenesis.^414^ This cytoplasmic localization of NPM occurred because of a frameshift in its last exon generated a new NES sequence that promoted its nuclear export.^414^ Understanding these different pathogenic mechanisms can help guide the development of precise medicines which specifically correct the localization of a particular cargo.

#### RNA export dysregulation and cancer

Similar to protein nuclear transport, RNA export is altered in many cancers. Human mRNA can be exported via XPO1/RanGTP-dependent pathways and NXT1/NXF1-dependent bulk export pathway.^415^ Multiple factors within these pathways are dysregulated across various cancers, such as XPO1, THO1, HuR, and eIF4E.^415–417^ In particular, ~ 30% of human cancers upregulate eIF4E levels, exporting a set of mRNAs containing eIF4E-sensitive elements through the XPO1 pathway.^418^ Many of these mRNAs encode oncogenes, including cyclin D1, NBS1, cMyc, and MDM2, leading to activation of proliferation pathways such as Akt.^419^ There are over a dozen clinical trials using the eIF4E inhibitor ribavirin on different cancers.^420^ The bulk mRNA export factor NXT1 was also identified as a genetic dependency in neuroblastoma and several pediatric cancers.^421^

Defects in pre-miRNA export are also observed across many cancers. Mature miRNAs are often downregulated in cancer, and several mechanisms are known to reduce miRNA levels.^422^ In a subset of human tumors with microsatellite instability, several XPO5-inactivating mutations trapped pre-miRNAs within the nucleus and reduced miRNA-target inhibition.^423^ In another study, ERK phosphorylation of XPO5 induces a conformational change in XPO5, making it unable to load pre-miRNA appropriately.^424^ XPO5 phosphorylation is associated with poor prognosis in liver cancer patients.^424^ Epigenetic change and abnormal XPO5 expression levels also impact miRNA expression and have profound effects on tumorigenesis.^132^

#### Nucleoporin fusions that alter transcription

In a wide array of hematopoietic malignancies, chromosome translocations often result in Nup98 oncogenic fusion proteins associated with poor prognosis.^425,426^ Fusion proteins typically include the N-terminal FG domain of Nup98 and the C-terminal domain of a partner protein such as HOXA9.^427^ Many fusion proteins physically interact with mixed lineage leukemia 1 (MLL1) and nonspecific lethal (NSL) histone-modifying complexes, an interaction that is critical for its leukemogenesis ability.^428,429^ These fusion proteins can upregulate the HOXA cluster gene and inhibit hematopoietic precursor differentiation (Fig. 8b).^385,425^ In acute myeloid leukemia, the loss of the direct transcriptional target CDK6 severely attenuated fusion-driven leukemogenesis.^430^ In addition to Nup98 fusions, fusions containing fragments of Nup214 or Tpr have also been observed in several cancers and can similarly drive cancer progression.^431–433^

The oncogenic property of fusions depends not only on fused domains that bind DNA or modify histones but also on the FG domain of Nup98.^385,434^ The FG domain is capable of liquid-liquid phase separation and is critical for puncta formation on chromatin.^354,372^ This property promotes binding between the fused domain and chromatin, generating a broad superenhancer-like binding pattern that potentiates transcriptional activation of proto-oncogenes.^354^

### Neurodegenerative diseases

Unlike cancers, nuclear transport is often impaired in neurodegenerative diseases (NDDs), including amyotrophic lateral sclerosis, frontotemporal dementia, Alzheimer’s disease, and Huntington’s disease.^435^ Cytoplasmic aggregation of RBP proteins such as TDP-43 and FUS is a hallmark of NDD.^436^ The microtubule-associated protein Tau is not known as an RBP but it is also capable of binding RNA and other RBPs, especially in disease.^437,438^ These RBP proteins are normally nuclear but are predominantly aggregated in the cytoplasm of diseased neurons.^439^ For example, mislocalization and aggregation of TAR-DNA binding protein 43 (TDP-43) is observed in ~98% of ALS cases.^440^ Similarly, cytoplasmic FUS aggregates are a pathological hallmark in a subset of patients with FTD or ALS. The loss of RBP nuclear function and gain of RBP cytoplasmic function are critical for the pathogenesis of NDD.^439^ Many studies indicate that impaired nuclear transport is responsible for RBP pathology and is a common factor in many NDDs,^350^ highlighting a promising area of research that could lead to the discovery of new therapies for NDDs.

#### The vicious cycle between nuclear transport impairment and RBP pathology

Impaired nuclear transport of RBP proteins is a major cause of RBP mislocalization. Some RBP cargo mutations that occur in neurodegenerative diseases promote accumulation and aggregation in the cytoplasm.^278^ For example, many FUS-NLS mutations impair TNPO1 binding, promoting cytoplasmic phase separation and stress granule partitioning of FUS.^343^ Phosphorylation or mutation of the NLS of TDP-43 disrupts the nuclear import and chaperone activity of Impα1/β1.^441^ Furthermore, many NTPs, including karyopherins and nucleoporins, are downregulated in NDD cells. Therefore, impaired nuclear transport is increasingly recognized as a pathogenic driver of neurodegeneration.^442^

**Fig. 9:**
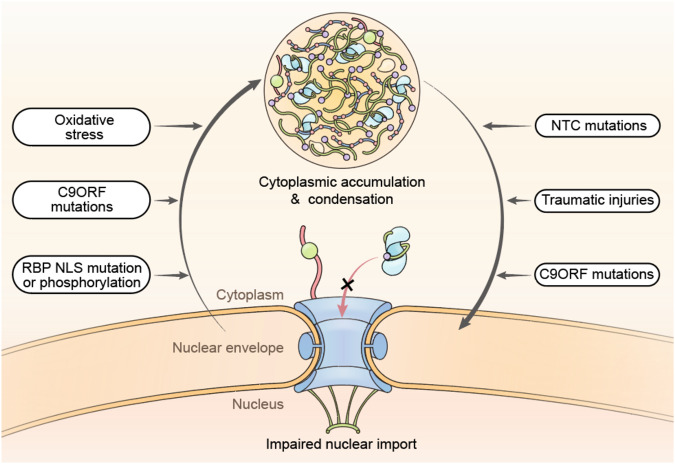
Vicious cycle between cytoplasmic condensation of RNA binding proteins (RBPs) and defective nuclear transport. Cytoplasmic condensation of RBPs (such as FUS and TDP-43) recruits many nuclear transport proteins (such as importins, nucleoporins, and Ran system proteins) into the condensates and disrupts normal nuclear transport (especially import). Impaired nuclear transport further leads to cytoplasmic accumulation of RBPs and excessive condensation. The vicious cycle can be triggered by genetic factors such as mutations or external factors such as chronic stress

The cytoplasmic aggregation of RBPs is not only a consequence of impaired nuclear transport but may in turn lead to defective nuclear transport. For example, cytoplasmic TDP-43 droplets may recruit and mislocalize importin-α, Nup62, RanGAP1, Ran, and Nup107, leading to inhibition of nuclear transport and eventual neuronal cell death.^443^ Likewise, pathogenic Tau can lead to NPC dysfunction by directly interacting with NPC components, causing their mislocalization.^444^ Therefore, current studies support the existence of a vicious cycle in NDD, i.e., the progressive deterioration of RBP localization and nuclear transport (Fig. 9).

#### Nuclear transport protein abnormalities in NDD

Karyopherins, especially importins, play an important role in the nuclear import of RBPs. For example, upregulation of Impα3 reduces the cytoplasmic accumulation of TDP-43 and mitigates behavioral deficits in mice.^445^ Alternatively, inhibition of protein nuclear export also suppresses neurodegeneration.^446^ In addition to their canonical role in nuclear import, importins sculpt cytoplasmic membraneless organelles and dissolve the ‘irreversible’ precipitates or beta-amyloid-like fibers formed by RBPs, as explained in section 4.2.2.^103^ Therefore, karyopherins exhibit two distinct roles in NDD, as mediators of RBP nuclear localization and as molecular chaperones that inhibit RBP aggregation, and karyopherin abnormalities can trigger the onset and progression of NDD.

Karyopherin abnormalities are widely observed in NDD.^83^ For example, protein levels of Impα1 and XPO2 are reduced in the frontal cortex of FTD patients, and Impβ1 is reduced in the spinal cord of ALS patients.^447,448^ Furthermore, karyopherins are often sequestered into stress granules and TDP-43 droplets in diseased neuron cells.^351,449^ Several patient-derived TNPO2 variants that impair RanGTP or cargo binding have been shown to be responsible for neurodevelopmental abnormalities.^450^ These observations are consistent with genetic perturbation studies, suggesting that karyopherin abnormalities may mediate NDD pathogenesis. Upregulating the expression levels of several aforementioned importins in neuronal cells of patients is a promising strategy for the treatment of those NDDs without RBP NLS mutations.

In addition to karyopherin abnormalities, loss of nuclear pores, nucleoporin aggregation, and altered nuclear morphology are some of the most prominent features across a variety of NDD cells and animal models.^451,452^ Through phase separation, aggregated TDP-43, FUS, and Tau mislocalize FG nucleoporins and trigger the structural and functional impairment of NPCs.^444,453^ For example, the nucleoporin Nup62 colocalizes with TDP-43 granules in diseased brain cells.^351,454^ Although importins can reduce these cytoplasmic condensates formed by RBPs and nucleoporins, they may be overwhelmed and trapped by these excessive biocondensates in diseased cells.^455^ Impaired mRNA export is often observed in NDD cells, as many FG nucleoporins are critical for mRNA export.^452,456^ Furthermore, specific nucleoporin mutations may lead to cell type-specific neurological disorders.^350^ For example, a homozygous splicing mutation in Nup133 causes Galloway-Mowat syndrome, highlighting the importance of nuclear transport in NDD.^457^ Nucleoporin alterations and the consequential loss of NPC function may lie upstream of TDP-43 mislocalization in NDD.^458^ Abnormalities in nucleoporins (such as Nup62, Nup93, Tpr, and Nup153) also impact non-neuron cells, contribute to aging and premature aging at the organism level.^459^

#### Other factors that may initiate the vicious cycle

Many studies have demonstrated that C9ORF72 hexanucleotide (GGGGCC) repeat expansion (HRE, either familial or sporadic) may act as an initiator of the vicious cycle. HRE, the most common genetic cause of ALS and FD, encodes proteins containing extra intrinsically disordered regions. One of the encoded poly-GR proteins tightly binds to Impα1, directly disrupting the nuclear import of endogenous cargoes.^460^ A mutant C9ORF72 can induce proteasome-mediated degradation of select nucleoporins.^461^ Mutations that generate extra intrinsically disordered regions in proteins such as huntingtin and ataxin1 can similarly initiate the vicious cycle.^462,463^ In addition to the protein, HRE RNA initiates a decrease in POM121, which may further lead to downregulation of seven additional nucleoporins.^464^ The HRE RNA can also sequester RanGAP1 and distort the RanGTP gradient, disrupting nuclear integrity and transport.^465^ Several components of nuclear transport can also effectively combat the toxicity of C9ORF72 HRE by means of nuclear import and anti-aggregation.^466^

In addition to genetic factors, external factors may initiate the vicious cycle. The formation of stress granules is a typical response of cells to a broad range of stresses. However, stress granule formation may sequester critical NTPs, like RBP biocondensates, thereby inhibiting nuclear transport.^467^ Therefore, constitutive oxidative stress throughout aging may persistently impair nuclear transport and lead to irreversible NDD. Likewise, a recent study using *Drosophila* demonstrated that traumatic injury leads to NPC defects, impairing the RanGTP gradient, and leading to cytoplasmic aggregation of Nup62 and TDP-43.^454^ This may explain why traumatic brain injury is a predisposing factor for several neurodegenerative diseases. Since cellular localization of a protein is determined by both its nuclear import and nuclear export, nuclear export inhibitors may be applied after a traumatic injury to prevent NDD. Based on two encouraging preclinical studies,^468,469^ it is worth further testing whether XPO1 inhibitors can clinically slow down or even reverse some of the discussed NDDs.

### Viral infection

Many viruses replicate in the nucleus of host cells and rely on the nuclear transport system for their nuclear entry. In addition, nuclear transport plays a role in other stages of the viral life cycle, such as uncoating and viral RNA export. Viruses have developed specific strategies to suppress host immune responses by targeting karyopherins, thereby avoiding clearance by the host. In addition to targeting karyopherins, viruses may inhibit or distort host nuclear transport by altering NPC integrity.

#### Exploitation of nuclear transport proteins to complete the viral infection cycle

Several RNA viruses and nearly all DNA viruses require access to the host cell nucleoplasm for replication.^470^ Following virus-cell fusion, a core consisting of capsid proteins (CA) surrounding the viral genomic DNA/RNA enters the cytoplasm of host cells.^471^ The NPC filament proteins Nup214 and Nup358 can bind to capsid proteins and are critical for NPC docking.^472^ Prior to genome import and replication in the nucleus, the core of most viruses must be uncoated by cytosolic host proteins such as karyopherins.^473^ TNPO1, which promotes the removal of M1 from the core by binding to a PY-NLS sequence in the matrix protein M1, is a common uncoating factor for influenza A virus (IAV) and human immunodeficiency virus type 1 (HIV-1).^107^

Most of these viruses utilize cellular nuclear import machinery for their nuclear entry.^474^ For example, IAV vRNP uses Impα7 for its nuclear import.^475^ Although viral mechanisms for crossing NPCs are complex and diverse, interaction with Impα/β1 is absolutely critical for nuclear entry of many viruses.^476^ The macrocyclic lactone ivermectin, which is reported to target IMPα/β1, has broad-spectrum activity against a variety of viruses, including HIV-1, DENV, ZIKV, West Nile virus (WNV), and SARS-CoV-2 (COVID-19).^387^ Our group, however, have observed no direct binding between ivermectin to IMPα1 or β1, nor inhibition of classical nuclear import using physiological relevant concentrations of ivermectin (unpublished). Through inhibition of the binding of NS5 and Impα1, N-(4-hydroxyphenyl) retinamide (4-HPR) has anti-ZIKV activity at low μM concentrations.^387^

Human retroviruses such as HIV and human T-cell leukemia virus type 1 (HTLV-1) require export of their intron-containing RNAs from the nucleus to the cytoplasm for translation and packaging. Two viral proteins, Rev and Rex, act as export adaptors to facilitate export of viral RNA through simultaneous binding of the viral RNA and the export factor XPO1.^477^ Inhibition of XPO1 thus results in sequestration of key viral accessory proteins and genomic materials in the host cell nucleus, thereby reducing the replication of viruses such as influenza, respiratory syncytial virus (RSV), and SARS-CoV.^478^ Clinical studies using the XPO1 inhibitor selinexor are currently in progress.^388^ Since XPO1 suppress inflammation and immune activation through the NFκB pathway, the application of XPO1 inhibitors in different cancers may also result in bacterial infection, a major cause of poor clinical outcomes.^478,479^ Therefore, combination with antibiotics should probably be considered in the treatment of cancers or viral infections.

#### Evasion of immune clearance by inhibiting protein nuclear import or mRNA nuclear export

As a defense mechanism, human cells upregulate the interferon response to combat viral infections. Typically, upon viral stimulation, IRF and STAT transcription factors are imported into the nucleus, subsequently transcribing and exporting an array of mRNAs encoding immune factors.^480^ Viruses have evolved different strategies to inhibit this process. Open reading frame 6 (ORF6) of SARS-CoV-2 binds to Impα1 to inhibit IRF3 nuclear import and the type I interferon response.^481^ Similarly, Ebola virus VP24 binds importin alpha proteins and inhibits STAT1 nuclear import, rendering cells refractory to IFNs.^187^ Zika virus NS2A protein induces degradation of Impα1 through chaperone-mediated autophagy.^482^ In contrast, the influenza virus NS1 protein blocks host mRNA nuclear export by directly interacting with the NXF1-NXT1 export machinery.^483^

Viruses can also disrupt the host immune response by targeting nucleoporins. For example, the 2A^pro^ protease of poliovirus and rhinovirus cleaves Nup62, Nup98, and Nup153.^484,485^ Alternatively, mengovirus and cardioviruses can inhibit nuclear transport through induction of hyperphosphorylation of nucleoporins such as Nup162, Nup35, and Nup214.^486,487^ Cytoplasmic mislocalization of NPC parts, such as Nup214, Nup358, and Nup62, is frequently observed in cells infected with different viruses.^472,488,489^ Moreover, ORF10 and ORF6 from several viruses repress host mRNA export by interacting with RAE1 and Nup98.^490,491^ These actions inhibit antiviral responses and may also prevent cell death to allow viral replication or induce NE leakage to permit viral genome entry into the nucleus.^472^ While the above findings are well documented, actual infections are often more complex and dependent on the specific virus and the infection stage.^492^

## Conclusion and perspectives

Due to the complexity and structural dynamics of NPCs, a complete atomic model has not been achieved thus far, despite enormous efforts. It is expected that with continuous improvements in resolution power and artificial intelligence, an almost complete atomic NPC structure will soon be obtained. Different conformations of NPCs may be obtained to illustrate how NPCs transit from one to another. While atomic models cannot be established for disordered regions, it should be possible to correctly understand their function in the broader context of structured regions. Such structural information is crucial for understanding the canonical function of NPCs, interpreting the pathogenic mechanisms of disease mutations, and developing targeted drugs for related diseases.

A few karyopherins, including XPO1 and classical nuclear import factors, are well studied; however, little is known about many other karyopherins. While some karyopherins (especially importins) are redundant in transporting certain cargoes, they do vary widely in terms of function and spatiotemporal expression. Furthermore, often selected karyopherins are reported to play key roles in different diseases. Therefore, it is important to study the structures, cargo recognition mechanisms, and affiliated pathways of each karyopherin. Furthermore, the development of potent and selective inhibitors has been limited to only a few karyopherins.^493^ The development of specific inhibitors is not only beneficial to basic research but may also provide new therapies for imminently threatening diseases.

In addition to their role in nuclear transport, nuclear transport proteins play roles in other cellular processes, such as mitosis, biomolecular condensate regulation, and transcription regulation. These functions share common fundamental principles with the nuclear transport function, including the interaction network and assembly principle, but can be completely distinguished from nuclear transport. For example, nuclear transport, mitosis, and gene transcriptional regulation of NTPs are important for tumorigenesis, while the nuclear transport and regulation of biomolecular condensate functions of NTPs are clearly involved in NDDs. Future studies involving NTPs should try to clarify which specific functions of an NTP are important for the phenotype or disease in question.

The nuclear transport system is a double-edged sword that keeps cells functioning properly. Generally, upregulation of nuclear transport may lead to cancer, and downregulation may lead to NDD. Viruses may either use nuclear transport to facilitate their replication or inhibit nuclear transport to evade immune surveillance. Therefore, while treating one disease, care must be taken to avoid causing another. For example, when treating tumors through inhibition of highly expressed NTPs, it is important to avoid triggering NDD development. Likewise, when upregulating importins to treat different NDDs, the risk of carcinogenesis should be considered. Tissue-specific targeting or delivery may be helpful in this regard.

## Supplementary information

Supplemental text
