# Supplementary material for: Nuclear transport proteins: structure, function and disease relevance
Source: Signal Transduct Target Ther. 2023 Nov 10;8:425. doi: 10.1038/s41392-023-01649-4 (PMC10636164; doi:10.1038/s41392-023-01649-4)
Supplement: Supplementary file 1 — Supplemental text [file 41392_2023_1649_MOESM1_ESM.docx]

# **Nuclear transport proteins: structure, function, and disease relevance**

Yang Yang^1#^, Lu Guo^1#^, Lin Chen^1#^, Bo Gong^2,3#^, Da Jia^4*^, Qingxiang Sun^1,5*^

^1^ Department of Pulmonary and Critical Care Medicine, Sichuan Provincial People's Hospital, School of Medicine, University of Electronic Science and Technology of China, Chengdu, China.

^2^ The Key Laboratory for Human Disease Gene Study of Sichuan Province and Department of Laboratory Medicine, Sichuan Academy of Medical Sciences & Sichuan Provincial People’s Hospital, University of Electronic Science and Technology of China, Chengdu, China.

^3^ Research Unit for Blindness Prevention of Chinese Academy of Medical Sciences (2019RU026), Sichuan Academy of Medical Sciences & Sichuan Provincial People’s Hospital, Chengdu, China.

^4^ Key Laboratory of Birth Defects and Related Diseases of Women and Children, Department of Pediatrics, West China Second University Hospital, State Key Laboratory of Biotherapy, Sichuan University, Chengdu, China.

^5^ Department of Pathology, State Key Laboratory of Biotherapy and Cancer Centre, West China Hospital, Sichuan University, and Collaborative Innovation Centre of Biotherapy, Chengdu, China.

**Supplemental text**

**Importin 4.** Importin 4 (abbreviated as either Imp4 or IPO4) is the primary nuclear import receptor for core histones H3 and H4, using its N-terminal half to clamp the globular H3-H4 domain and the H3 αN helix.^1^ It interacts with the LPPRS(G/P)P linear motifs of many proteins, but the interaction mechanism is unclear.^2^ IPO4 is also responsible for nuclear import of the vitamin D receptor and the transcription factor CEBPD.^3-5^ Many IPO4 cargoes are involved in DNA metabolism and chromosome organization.^6^

**Importin 5 and RanBP6.** Human Importin 5 (IPO5, Imp5, also known as RanBP5, or KPNB3) has been demonstrated to interact with a linear KP(K/Y)LV motif, somewhat different from the recognition motif of yeast IPO5.^2,7^ This protein is responsible for the import of ribosomal proteins, histone H3, influenza A virus RNA polymerase subunit PB1, and Down syndrome cell adhesion molecule DSCAM.^8-11^ Many IPO5 cargoes function in ribosome biosynthesis.^6^ The closely related RanBP6 (79% identical) has been incompletely characterized, except that it mediates nuclear translocation of signal transducer and activator of transcription 3 (STAT3) to suppress glioblastoma.^12^

**Importin 7 and Importin 8.** Importin 7 (IPO7 or Imp7) imports histone H1, HIV reverse transcription complex (RTC), the TGF-β signaling component Smad1, and the Hippo signaling pathway effector Yap1.^13-16^ The C-terminal FxFG motif of IPO7 recruits Impβ1 to form a cradle for the binding and nuclear import of histone H1.^17^ IPO7 is required for nuclear import of plasmids in a DNA sequence-specific manner, but whether IPO7 directly recognizes DNA is unknown.^18^ Importin 8 (IPO8 or Imp8) shares 64% sequence identity with IPO7 and is less well characterized. It mediates the nuclear import of Smad proteins, eukaryotic translation initiation factor eIF4E, and mature microRNAs.^19-22^ Biallelic IPO8 mutations impair Smad import, leading to cardiovascular defects, skeletal abnormalities, and immune dysregulation.^19,23^ Both IPO7 and IPO8 import a range of mRNA splicing factors. However, IPO7 imports more small nuclear ribonucleoproteins (snRNPs) and high-mobility group (HMG) proteins, while IPO8 imports an increased number of ribosomal proteins.^6^

**Importin 9.** Importin 9 (IPO9, Imp9, also known as RanBP9) imports the transcription factor AT-rich interacting domain 3A (ARID3A), the major isoform of phosphofructokinase (PFKP), and histones H2A and H2B.^24-26^ Structural analysis revealed that IPO9 uses its convex surface to wrap core globular regions of H2A-H2B.^25^ The nuclear import of actin by IPO9 is required for maximal transcriptional activity.^27^ Many IPO9 cargoes are ribosomal proteins or mRNA splicing factors.^6^

**Importin 11.** Importin 11 (IPO11, Imp11, also known as RanBP11) mediates nuclear import of the transcription factors BZW1/2, β-catenin, the ubiquitin-conjugating enzyme UbcM2, and tumor suppressor PTEN.^28-32^ Depletion of IPO11 results in PTEN degradation within the cytoplasm and lung tumor development in mice.^31^ The mechanism of cargo recognition of IPO11 is currently lacking. IPO11 cargoes identified by proteomic analysis are linked to both developmental processes and nuclear division.^6^

**Exportin t.** Exportin t (XPOT) is a dedicated nuclear export receptor for mature tRNA.^33^ XPOT wraps around RanGTP and the tRNA stem while binding to the 5' and 3' ends of the tRNA to ensure that only mature tRNAs are exported.^34^ Other XPOT cargoes have not been reported, and XPOT appears to be nonessential for all tested organisms, as tRNAs are exported by multiple pathways.^35^

**Exportin 6.** Exportin 6 (XPO6) exports nuclear actin and profilin-1.^36-38^ However, the mechanism of their interaction and whether XPO6 exports more cargo are unclear. In breast cancer, XPO6 overexpression leads to decreased levels of nuclear profilin-1 as well as enhanced MYC transcription.^38^ XPO6 also participates in the transport of rice stripe virus into exosomes.^39^

**References**

1 Bernardes, N. E. *et al.* Structure of IMPORTIN-4 bound to the H3-H4-ASF1 histone-histone chaperone complex. *Proc Natl Acad Sci U S A*. **119**, e2207177119, (2022).

2 Panagiotopoulos, A. A. *et al.* Recognition motifs for importin 4 [(L)PPRS(G/P)P] and importin 5 [KP(K/Y)LV] binding, identified by bio-informatic simulation and experimental in vitro validation. *Comput Struct Biotechnol J*. **20**, 5952-5961, (2022).

3 Wang, J. *et al.* CCAAT/enhancer binding protein delta (C/EBPdelta, CEBPD)-mediated nuclear import of FANCD2 by IPO4 augments cellular response to DNA damage. *Proc Natl Acad Sci U S A*. **107**, 16131-16136, (2010).

4 Miyauchi, Y. *et al.* Importin 4 is responsible for ligand-independent nuclear translocation of vitamin D receptor. *J Biol Chem*. **280**, 40901-40908, (2005).

5 Zhou, Y. *et al.* Inhibiting Importin 4-mediated nuclear import of CEBPD enhances chemosensitivity by repression of PRKDC-driven DNA damage repair in cervical cancer. *Oncogene*. **39**, 5633-5648, (2020).

6 Kimura, M. *et al.* Extensive cargo identification reveals distinct biological roles of the 12 importin pathways. *Elife*. **6**, (2017).

7 Kobayashi, J. & Matsuura, Y. Structural basis for cell-cycle-dependent nuclear import mediated by the karyopherin Kap121p. *J Mol Biol*. **425**, 1852-1868, (2013).

8 Mohl, G. *et al.* Novel influenza inhibitors designed to target PB1 interactions with host importin RanBP5. *Antiviral Res*. **164**, 81-90, (2019).

9 Sachse, S. M. *et al.* Nuclear import of the DSCAM-cytoplasmic domain drives signaling capable of inhibiting synapse formation. *EMBO J*. **38**, (2019).

10 Jakel, S. & Gorlich, D. Importin beta, transportin, RanBP5 and RanBP7 mediate nuclear import of ribosomal proteins in mammalian cells. *EMBO J*. **17**, 4491-4502, (1998).

11 Pardal, A. J. & Bowman, A. J. A specific role for importin-5 and NASP in the import and nuclear hand-off of monomeric H3. *Elife*. **11**, (2022).

12 Oldrini, B. *et al.* EGFR feedback-inhibition by Ran-binding protein 6 is disrupted in cancer. *Nat Commun*. **8**, 2035, (2017).

13 Garcia-Garcia, M. *et al.* Mechanical control of nuclear import by Importin-7 is regulated by its dominant cargo YAP. *Nat Commun*. **13**, 1174, (2022).

14 Xu, L. *et al.* Msk is required for nuclear import of TGF-beta/BMP-activated Smads. *J Cell Biol*. **178**, 981-994, (2007).

15 Jakel, S. *et al.* The importin beta/importin 7 heterodimer is a functional nuclear import receptor for histone H1. *EMBO J*. **18**, 2411-2423, (1999).

16 Fassati, A. *et al.* Nuclear import of HIV-1 intracellular reverse transcription complexes is mediated by importin 7. *EMBO J*. **22**, 3675-3685, (2003).

17 Ivic, N. *et al.* Fuzzy Interactions Form and Shape the Histone Transport Complex. *Mol Cell*. **73**, 1191-1203 e1196, (2019).

18 Miller, A. M., Munkonge, F. M., Alton, E. W. & Dean, D. A. Identification of protein cofactors necessary for sequence-specific plasmid DNA nuclear import. *Mol Ther*. **17**, 1897-1903, (2009).

19 Ziegler, A. *et al.* Bi-allelic variants in IPO8 cause a connective tissue disorder associated with cardiovascular defects, skeletal abnormalities, and immune dysregulation. *Am J Hum Genet*. **108**, 1126-1137, (2021).

20 Yao, X., Chen, X., Cottonham, C. & Xu, L. Preferential utilization of Imp7/8 in nuclear import of Smads. *J Biol Chem*. **283**, 22867-22874, (2008).

21 Wei, Y. *et al.* Importin 8 regulates the transport of mature microRNAs into the cell nucleus. *J Biol Chem*. **289**, 10270-10275, (2014).

22 Volpon, L. *et al.* Importin 8 mediates m7G cap-sensitive nuclear import of the eukaryotic translation initiation factor eIF4E. *Proc Natl Acad Sci U S A*. **113**, 5263-5268, (2016).

23 Van Gucht, I. *et al.* A human importin-beta-related disorder: Syndromic thoracic aortic aneurysm caused by bi-allelic loss-of-function variants in IPO8. *Am J Hum Genet*. **108**, 1115-1125, (2021).

24 Gao, X. *et al.* Nuclear PFKP promotes CXCR4-dependent infiltration by T cell acute lymphoblastic leukemia. *J Clin Invest*. **131**, (2021).

25 Padavannil, A. *et al.* Importin-9 wraps around the H2A-H2B core to act as nuclear importer and histone chaperone. *Elife*. **8**, (2019).

26 Liao, T. T. *et al.* let-7 Modulates Chromatin Configuration and Target Gene Repression through Regulation of the ARID3B Complex. *Cell Rep*. **14**, 520-533, (2016).

27 Dopie, J. *et al.* Active maintenance of nuclear actin by importin 9 supports transcription. *Proc Natl Acad Sci U S A*. **109**, E544-552, (2012).

28 Nachmias, B. *et al.* IPO11 regulates the nuclear import of BZW1/2 and is necessary for AML cells and stem cells. *Leukemia*. **36**, 1283-1295, (2022).

29 Ni, H. *et al.* The nuclear transporter importin-11 regulates the Wnt/beta-catenin pathway and acts as a tumor promoter in glioma. *Int J Biol Macromol*. **176**, 145-156, (2021).

30 Mis, M. *et al.* IPO11 mediates betacatenin nuclear import in a subset of colorectal cancers. *J Cell Biol*. **219**, (2020).

31 Chen, M. *et al.* The nuclear transport receptor Importin-11 is a tumor suppressor that maintains PTEN protein. *J Cell Biol*. **216**, 641-656, (2017).

32 Plafker, S. M. & Macara, I. G. Importin-11, a nuclear import receptor for the ubiquitin-conjugating enzyme, UbcM2. *EMBO J*. **19**, 5502-5513, (2000).

33 Arts, G. J., Fornerod, M. & Mattaj, I. W. Identification of a nuclear export receptor for tRNA. *Curr Biol*. **8**, 305-314, (1998).

34 Cook, A. G., Fukuhara, N., Jinek, M. & Conti, E. Structures of the tRNA export factor in the nuclear and cytosolic states. *Nature*. **461**, 60-65, (2009).

35 Chatterjee, K., Marshall, W. A. & Hopper, A. K. Three tRNA nuclear exporters in S. cerevisiae: parallel pathways, preferences, and precision. *Nucleic Acids Res*. **50**, 10140-10152, (2022).

36 Stuven, T., Hartmann, E. & Gorlich, D. Exportin 6: a novel nuclear export receptor that is specific for profilin.actin complexes. *EMBO J*. **22**, 5928-5940, (2003).

37 Chatzifrangkeskou, M. *et al.* RASSF1A is required for the maintenance of nuclear actin levels. *EMBO J*. **38**, e101168, (2019).

38 Zhu, C. *et al.* Cancer-associated exportin-6 upregulation inhibits the transcriptionally repressive and anticancer effects of nuclear profilin-1. *Cell Rep*. **34**, 108749, (2021).

39 Lu, H. *et al.* Key role of exportin 6 in exosome-mediated viral transmission from insect vectors to plants. *Proc Natl Acad Sci U S A*. **119**, e2207848119, (2022).
